# Multi-Targeted Therapeutic Mechanisms of Huangqi Guizhi Wuwu Decoction Against Rheumatoid Arthritis: An Integrated Approach Combining Serum Pharmacochemistry, Network Pharmacology, Metabolomics, and Experimental Validation

**DOI:** 10.3390/ph19020236

**Published:** 2026-01-29

**Authors:** Zihua Xu, Zhenshu Li, Jiameng Qu, Chen Liang, Yingshi Zhang, Qingchun Zhao, Qing Li

**Affiliations:** 1 School of Pharmacy, Shenyang Pharmaceutical University, Shenyang 110016, China; xuzihua-668585@163.com (Z.X.); qujiameng0519@163.com (J.Q.); liang0326chen@163.com (C.L.); 2 Graduate School of China Medical University, China Medical University, Shenyang 110122, China; lzs000905@163.com; 3 Department of Clinical Pharmacy, Shenyang Pharmaceutical University, Shenyang 110016, China; zhangyingshi526@163.com

**Keywords:** HGWD, RA, multi-targeted therapeutic mechanisms, network pharmacology, metabolomics

## Abstract

**Background**: The pathogenesis of rheumatoid arthritis (RA) is closely related to multiple disorders in the immune and metabolic systems, which indicates that a multi-target therapy strategy may have advantages over traditional single-target therapy. Huangqi Guizhi Wuwu Decoction (HGWD), as a classic traditional Chinese medicine formula that has been used to treat RA in clinic, is a potential source of multi-target natural medicine. However, its active components and mechanism of action still need further research. **Methods**: This study combined serum pharmacochemistry, non-targeted metabonomics, network pharmacology, and experimental verification and comprehensively analyzed the therapeutic mechanism and pharmacodynamic basis of HGWD. **Results**: Through HPLC-Q-TOF-MS/MS, a total of 99 chemical components were identified. Among them, 25 prototype compounds were absorbed into the systemic circulation. The study of network pharmacology indicates that these compounds are concentrated in TNF, IL-17, and MAPK signaling pathways. In collagen-induced arthritis rats, HGWD can effectively alleviate joint inflammation, inhibit the production of pro-inflammatory cytokines (TNF-α, IL-1β, IL-6, IL-17), block the activation of the MAPK pathway, and restore 13 abnormal metabolic markers related to lipid and amino acid metabolism. In addition, the researchers identified and verified the combination of four active components (calycosin, paeoniflorin, 6-gingerol, and formononetin) in vitro, and its anti-inflammatory and anti-migration activities were equivalent to or stronger than those of the complete extract. Pharmacokinetic analysis also confirmed that these components were fully exposed in vivo. **Conclusions**: These findings reveal the mechanism of multi-component therapy of HGWD and identify the potential bioactive components, which can be used to develop multi-target therapeutic drugs for RA based on natural products.

## 1. Introduction

Rheumatoid arthritis (RA) is a very common chronic systemic autoimmune disease, which makes cartilage slowly deteriorate. Over time, the membrane in the joint becomes inflamed all the time, the bone is destroyed, and this destruction is irreversible, which may eventually lead to joint deformation and the inability to move normally [1,2]. The onset of RA is caused by a complex imbalance in the immune system. The main mechanism involves the disruption of the Th17/Treg cell balance and the abnormal excessive production of pro-inflammatory cytokines (such as TNF-α, IL-6, IL-17, etc.). These factors jointly drive the pathological activation of synovial fibroblasts (FLS), enabling them to acquire an aggressive phenotype, which directly leads to the progressive destruction of articular cartilage and bone [1,3]. This multi-faceted pathogenesis indicates that RA is extremely complex, and its treatment cannot rely on a single target.

At present, the international treatment guidelines suggest that anti-rheumatic drugs (DMARDs) to improve the condition should be used as the basis for the treatment of RA. Among them, methotrexate (MTX) is the first drug to be used [4,5]. However, the long-term efficacy of MTX is often limited by the dose-limiting toxicity of the drug, and these adverse reactions can affect multiple systems such as the nervous, digestive, and hematopoietic system [6,7]. Biological DMARDs, especially TNF inhibitors, have significantly transformed the treatment approach for RA. However, they can significantly increase the risk of severe infections, and therefore cannot be used in patients with hepatitis B, hepatitis C, tuberculosis, or other serious complications [8,9]. Moreover, the high cost of biological agents is the main obstacle restricting their wide accessibility, imposing a heavy burden on global patients and the healthcare system [10,11]. These unmet clinical need highlights the urgency of developing new treatment strategies that offer both excellent efficacy, higher safety, and better cost-effectiveness.

Under this background, multi-target natural products extracted from Traditional Chinese Medicine (TCM) have attracted significant attention as potential therapeutic strategies for the treatment of RA. Different from traditional single-target drugs, the multi-component system of a TCM formula can regulate multiple pathological processes, which may bring synergistic therapeutic effects and reduce toxicity [12,13,14]. The multi-component, multi-target synergistic treatment strategy of TCM is highly consistent with the complex pathological mechanism of RA, which may be used as a promising treatment. According to TCM theory, RA belongs to the category of “Bi” syndrome. Traditionally, it is treated with herbal formulas aimed at nourishing the liver and kidneys, replenishing qi, nourishing the blood, promoting circulation, and dissolving blood stasis to alleviate pain.

Among those classic prescriptions of TCM, Huangqi Guizhi Wuwu Decoction (HGWD) was first recorded in the ancient medical classics synopsis of the Golden Chamber (“Jin Kui Yao Lue”). The prescription has been used to relieve the symptoms of arthritis and joint pain for hundreds of years. HGWD comprises five medicinal herbs: Huangqi (*Astragalus membranaceus*), Guizhi (*Cinnamomi ramulus*), Baishao (*Paeoniae radix* Alba), Shengjiang (*Zingiberis rhizoma* Recens), and Dazao (*Jujubae fructus*). Currently, it has been applied in the clinical treatment of chemotherapy-induced peripheral neuropathy and autoimmune arthritis [15,16,17,18]. In the TNF transgenic mouse model, HGWD showed excellent therapeutic effect, which can effectively reduce joint inflammation and improve cardiopulmonary complications [18]. Although the preliminary network pharmacology and molecular docking research predicted its multi-target mechanism of action on RA, the systematic identification of its active ingredients and the therapeutic mechanism have not been clarified [19]. Therefore, to explore the relationship between the complex chemical components of HGWD and its overall efficacy is the fundamental premise to promote the standardization of its clinical application and further understand its mechanism.

Network pharmacology, with its holistic and systematic analytical paradigm, has become the core methodology for studying the complex systems of TCM, which involve multiple components and multiple targets [20]. When combined with serum pharmacochemistry, this integration strategy provides a powerful basis for identifying the real active substances from the complex chemical matrix of herbal preparations [21]. In addition, pharmacokinetic studies can clarify the in vivo process of active ingredients and its relationship between blood drug concentration and efficacy. However, it should be noted that the parameters may vary depending on the species and the pathological state of the organism [22]. In addition, metabonomics can systematically reveal the endogenous metabolic disorders under disease and drug intervention, and clarify its metabolic regulation mechanism. Therefore, the integrated systematic research strategy integrating serum pharmacochemistry, pharmacokinetics, and metabonomics provides a strong methodological support for identifying the active ingredients of TCM, clarifying its mechanism of action, and confirming the pharmacodynamic material basis of TCM formulations.

This study aims to systematically elucidate the pharmacological basis and multi-target mechanism of HGWD in treating RA by integrating techniques such as serum pharmacology, network pharmacology, and metabolomics. Firstly, the blood components were screened based on serum pharmacochemistry, and their targets and pathways were predicted by network pharmacology. Subsequently, the anti-arthritis effect of HGWD and its regulatory effect on inflammatory factors and signaling pathways were evaluated in a CIA rat model and verified in vitro in TNF-α induced MH7A cells and LPS stimulated RAW264.7 cells. Finally, the dynamic process of key components was revealed by pharmacokinetic analysis in vivo. This study provides a systematic experimental basis for the clinical application of HGWD and deepens the understanding of the mechanism of action of a multi-target TCM compound.

## 2. Results

### 2.1. Chemical Profiling of HGWD and Identification of Serum-Absorbed Constituents

To find out what chemical components are in HGWD, we used a machine called HPLC-Q-TOF-MS/MS to check its decoction, using both positive and negative ion modes. The total ion chromatogram (TIC) can be seen in Figure S4 in the Supplementary Materials. Through the accurate molecular weight, isotope distribution, and MS/MS fragment spectrum (analyzed by PeakView^®^ software, version 2.2, Sciex), 99 chemical components were preliminarily identified and classified into 13 categories: flavonoids (16 species), monoterpenes (19 species), triterpenoids (12 species), phenylpropanoids (8 species), amino acids (7 species), alkaloids (5 species), sugars (5 species), phenols (4 species), nucleosides (4 species), and gingerols. Detailed mass spectrometry data of all identified components are summarized in Table S12.

According to the theory of serum pharmacochemistry, orally administered herbal medicines exert their therapeutic effects primarily through constituents that are absorbed into the bloodstream and reach target tissues at bioactive concentrations [23]. To identify potential anti-arthritis active components in HGWD, this study compared the plasma samples of the HGWD administration group with the control group. Blood samples were collected at 1, 2, 4, and 6 h after administration. Through comparative analysis, the components detected only in the plasma of the HGWD group were identified as the “parent compounds” that were absorbed. Based on this, a total of 25 parent compounds were identified, mainly including flavonoids (such as calycosin, formononetin, isomicronulatol), monoterpenoids (such as paeoniflorin, albiflorin, mudanpioside), gingerols (such as 6-gingerol, 6-shogaol), phenylpropanoids (such as cinnamic acid, 6-methylcoumarin), organic acids (such as protocatechuic acid, azelaic acid), and amino acids (such as L-L-phenylalanine, L-tryptophan) and so on. The mass spectrometric characteristics, retention times, and herbal origins of these serum-absorbed constituents are summarized in Table 1, with the TICs of the plasma samples provided in the Supplementary Materials. Notably, the 25 absorbed compounds originated from all five constituent herbs of HGWD, with *Astragali radix* and *Paeoniae radix* Alba contributing the largest number of bioavailable constituents. Among these, flavonoids, such as calycosin and formononetin, from *Astragali radix*, monoterpene glycosides, including paeoniflorin and albiflorin, from *Paeoniae radix* Alba, and gingerols, such as 6-gingerol, from *Zingiberis rhizoma* Recens have been previously described to have anti-inflammatory and immunomodulatory effects. The identification of these pharmacologically relevant compounds in the systemic circulation provided a rational basis for subsequent network pharmacology analysis to predict their potential targets and mechanisms in RA treatment.

### 2.2. HGWD Alleviated Arthritis Progression and Disease Severity of CIA Rats

**Figure 1:**
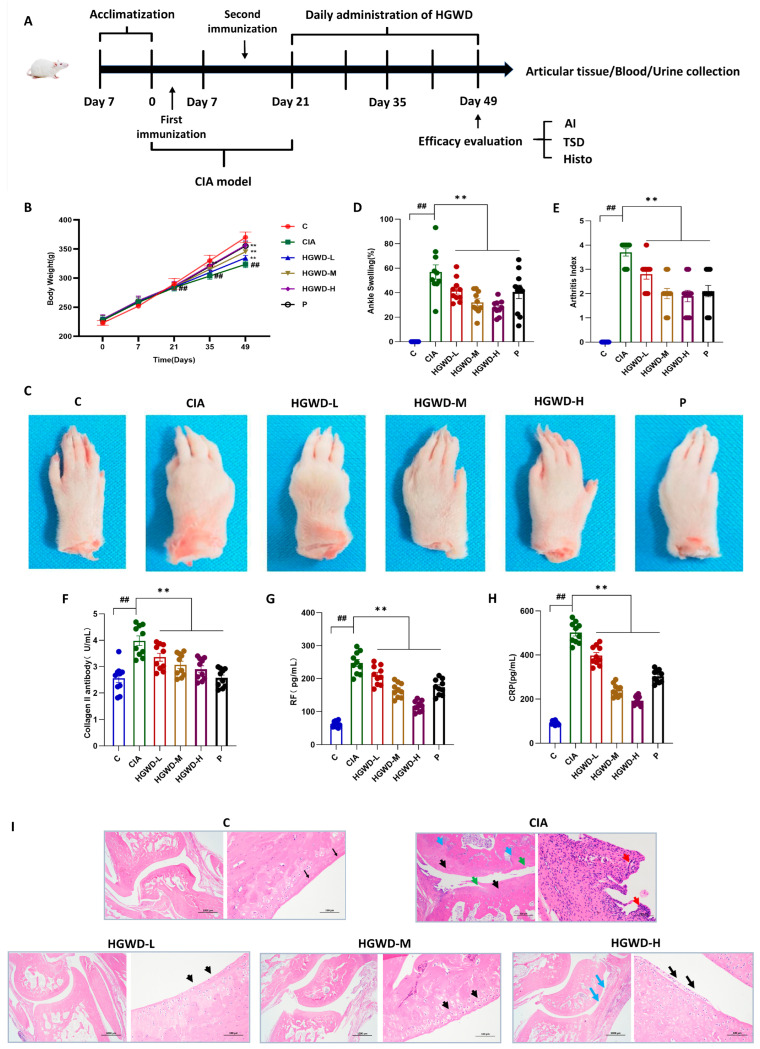
HGWD ameliorates disease severity and systemic inflammation in CIA rats. (**A**) Rats were divided into the following groups: Control (C), Model (CIA), HGWD-Low (HGWD-L), HGWD-Medium (HGWD-M), HGWD-High (HGWD-H), and methotrexate (MTX, P). (**B**) Body weight changes over time (*n* = 8); (**C**) representative photographs of hind paw arthritis; (**D**) the percentage of with ankle swelling (*n* = 8); (**E**) clinical arthritis index scores (*n* = 8); (**F**–**H**) serum levels of the following: Collagen II antibody (**F**), rheumatoid factor (RF, (**G**)), and C-reactive protein (CRP, (**H**)); and (**I**) Histopathological images of ankle joint sections (H&E staining). Data are presented as mean ± SD. ** *p* < 0.01 vs. the CIA group; ## *p* < 0.01 vs. the Control.

To estimate the therapeutic potential of HGWD against RA, a CIA rat model was created, and the experimental workflow is illustrated in Figure 1A. Throughout the 28-day treatment period, all rats exhibited a gradual increase in body weight; however, the Model (M) group consistently showed the lowest body weight gain compared to the Control (C), reflecting the systemic catabolic effects associated with CIA. It is worth noting that HGWD treatment can reverse the above pathological trend in a dose-dependent manner. The effect of the high-dose HGWD treatment group on weight recovery was similar to that of the positive control drug methotrexate group (Figure 1B). To further evaluate its anti-arthritis effect, this study observed the degree of paw swelling and the clinical arthritis score. Representative photos of the limbs after the experiment showed that the joints of the Model group mice were significantly swollen and red, while the symptoms were significantly alleviated after HGWD treatment (Figure 1C). Quantitative analysis indicated that the paw swelling rate of the Model group (Figure 1D) and the arthritis index (Figure 1E) were significantly higher than those of the Control group (*p* < 0.01). Oral administration of HGWD could reduce these two indicators in a dose-dependent manner, and the effect of the high-dose group was similar to that of the MTX group.

Since RA is characterized by the production of harmful autoantibodies that damage joints, the level of autoantibodies in serum was subsequently detected [35]. As expected, the serum concentrations of anti-type II collagen antibody (anti-CII, Figure 1F), rheumatoid factor (RF, Figure 1G) and C-reactive protein (CRP, Figure 1H) in group M were much higher than those in group C (*p* < 0.01). HGWD treatment significantly suppressed the levels of these inflammatory and autoimmune markers dose-dependently (*p* < 0.01), suggesting its potential immunomodulatory effects on the humoral immune response in CIA rats. Histopathological examination of the ankle joint section further confirmed the therapeutic effect of HGWD (Figure 1I). Group M showed typical pathological features of RA, such as obvious synovial hyperplasia, invasive cell infiltration, cartilage surface erosion (accompanied by crack formation), chaotic chondrocyte arrangement, and subchondral bone destruction. In contrast, these histopathological abnormalities were markedly attenuated in the HGWD-treated groups, with the HGWD-H group showing near-normal joint architecture. Collectively, these findings demonstrated that HGWD effectively ameliorated arthritis progression and disease severity in CIA rats, providing a solid pharmacological foundation for subsequent investigations into its active constituents and underlying mechanisms.

### 2.3. Network Pharmacology Analysis Revealed Potential Targets and Pathways of HGWD Against RA

**Figure 2:**
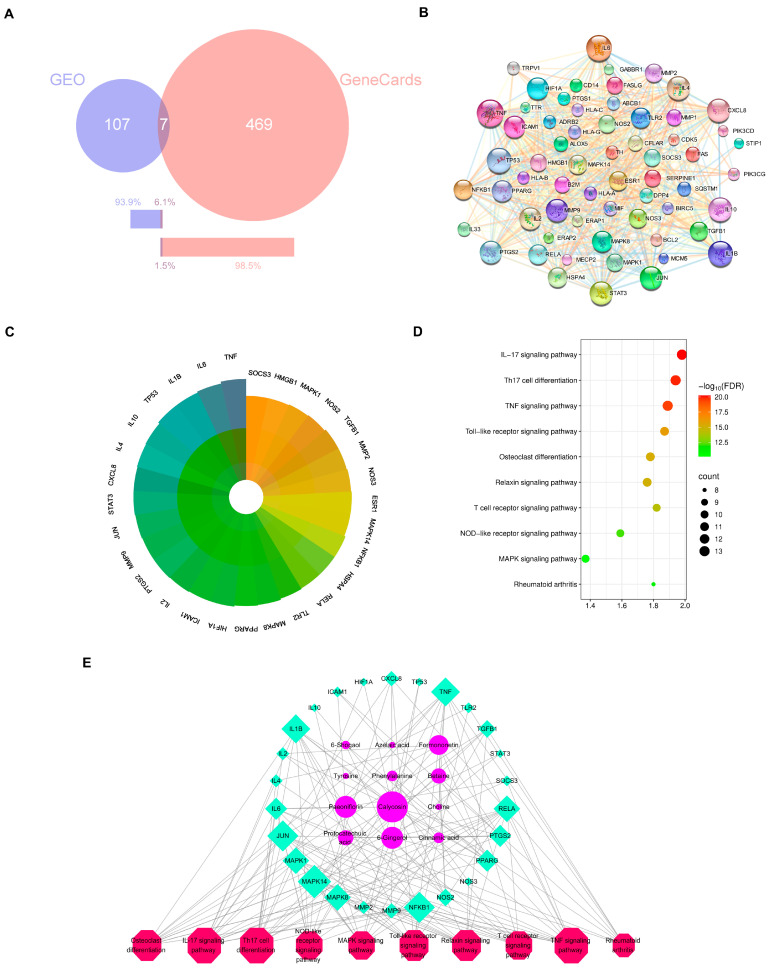
Network pharmacology of serum-absorbed constituents of HGWD and RA-related targets. (**A**) Venn diagram showing the intersection of HGWD compound targets and RA-related targets. (**B**) The PPI network of 61 disease–drug intersecting targets. (**C**) The PPI core targets of HGWD for the treatment of RA. Core targets are defined with the degree value > 21.4. The darker the color, the higher the degree and the more importance. (**D**) The top 10 pathways for KEGG enrichment analysis for the core targets. (**E**) Interaction network of active ingredient—therapeutic target—KEGG pathways. The pink octagons represents active ingredients, the blue circles represent the targets of HGWD, and the green quadrilaterals represent the pathways. The larger the area, the more important the target.

To explain the molecular mechanisms of the anti-arthritic effects of HGWD, network pharmacology analysis was performed based on the 25 serum-absorbed prototype compounds identified in Section 2.1. The potential targets of these compounds were predicted via multiple databases, including Swiss Target Prediction, HERB, and SEA, yielding 469 putative targets. Meanwhile, RA-related disease targets were acquired from the GEO and GeneCards databases, with the RA-related dataset obtained from the GEO database (accession number: GSE68689). “Rheumatoid Arthritis” was used as the search term in both databases, with the species restricted to Homo sapiens. The retrieved genes were first standardized to Gene Symbols, and then the gene lists from the two databases were merged. Duplicate entries were removed using Excel, resulting in 583 disease-associated genes. By intersecting the compound targets with disease targets, 61 overlapping targets were identified as prospective therapeutic targets of HGWD for RA treatment (Figure 2B). To identify the core targets with higher biological significance, a protein–protein interaction (PPI) network was created via the STRING. Topological analysis illustrated that targets with degree values greater than the average (21.4) were more likely to play pivotal roles in the pharmacological network. Accordingly, 29 core targets were selected for further analysis, including TNF, IL-6, IL-1β, PTGS2, MMP9, MAPK1, MAPK3, JUN, and STAT3, among others (Figure 2C). These targets are well-established mediators of inflammation, immune regulation, and joint destruction in RA pathogenesis, suggesting that HGWD may exert its therapeutic effects through modulating multiple key nodes in the RA-associated biological network.

Then we did KEGG enrichment analysis to find out the signal pathways related to those 29 core targets. We regarded the pathways with FDR < 0.05 as really important, and then according to the number of targets contained in them and related literature reports, we select the top ranked pathways. As shown in Figure 2D, these most important pathways include Th17 cell differentiation, osteoclast differentiation, and TNF, IL-17, Toll-like receptor, MAPK, T-cell receptor, NF-κB, and relaxin signaling pathways. In particular, the differentiation pathways of TNF, IL-17, and Th17 cells are very important in the RA-specific inflammatory response and autoimmune response, while the MAPK signaling pathway, like the downstream executor, is responsible for transmitting signals and influencing cytokine production and cellular response [36,37,38,39]. These findings suggest that HGWD may help improve RA by regulating the differentiation of immune cells and the transmission of inflammatory signals together.

In order to understand the relationship between active compounds, their therapeutic targets, and related pathways, Cytoscape 3.9.0 software was used to draw the “compound target pathway” (CTP) network diagram (Figure 2E). This network includes 12 compounds, 26 targets, and 10 pathways. The way they are connected with each other just reflects the characteristics of HGWD’s “multi-component and multi-target”. Among the 12 active compounds, calycosin, paeoniflorin, 6-gingerol, and formononetin have the highest “degree value”, which shows that they play a core role in this pharmacological network. These four compounds were therefore selected as representative active constituents for subsequent experimental validation. Based on the network pharmacology predictions, Th17 cell differentiation, and the IL-17 and TNF pathways were prioritized as key mechanistic targets for in vivo and in vitro verification in the following sections.

### 2.4. Plasma Metabolomics Analysis Revealed RA-Associated Metabolic Disturbances and the Regulatory Effects of HGWD

**Figure 3:**
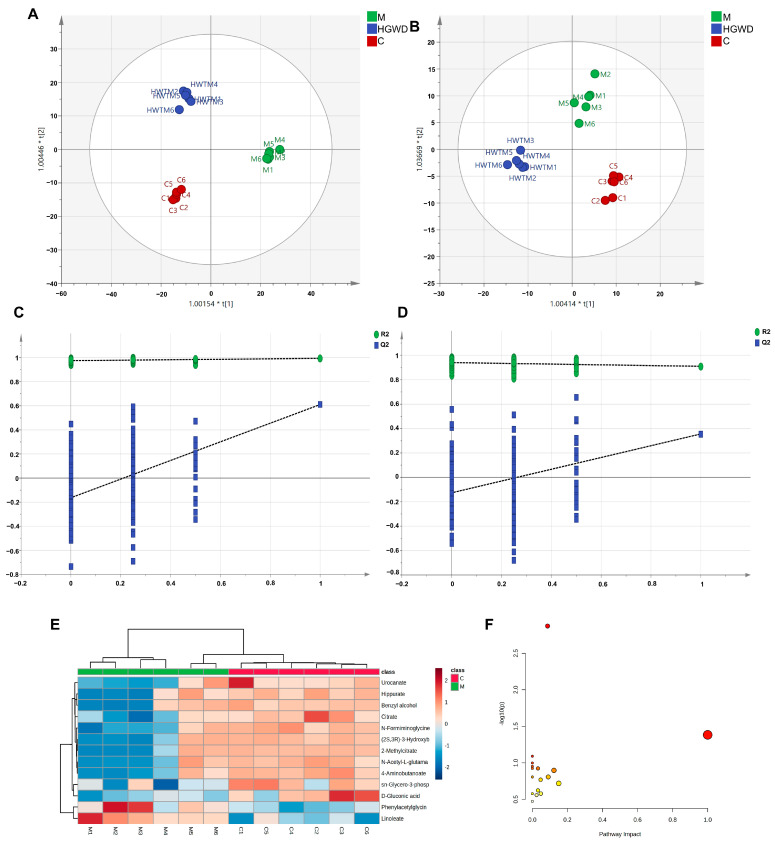
Non-targeted metabolomics analysis of plasma from CIA rats and the modulatory effect of HGWD. OPLS-DA score plots in positive (**A**) and negative (**B**) ion modes and corresponding permutation tests (**C**,**D**) are shown. (**E**) Heatmap of 13 potential plasma biomarkers in the control (C) and model (M) groups. (**F**) Pathway analysis of perturbed metabolic pathways depending on the identified biomarkers.

To investigate systemic metabolic changes in RA and the modulatory effects of HGWD, untargeted metabolomics was performed on plasma from the Control (C), Model (M), and HGWD-M groups using HPLC-Q-TOF-MS/MS. Figure S5 presents TICs in positive and negative ion modes. Orthogonal partial least squares-discriminant analysis (OPLS-DA) was used to visualize metabolic differences, and OPLS-DA models were further created to maximize group separation and identify discriminating metabolites. Figure 3A,B illustrates that clear separation was detected between the C and M groups, indicating significant metabolic perturbations induced by CIA. It is worth noting that the HGWD-M group tends to move closer to the C group, indicating that the treatment with HGWD partially restored the disturbed metabolic state. Permutation tests (*n* = 200) verified the reliability and effectiveness of the OPLS-DA model, and no fitting signs (Figure 3C,D) were found.

Potential biomarkers were screened according to a series of strict criteria: in the OPLS-DA model, the variable importance in projection (VIP) was greater than 1.0, the fold change (FC) was greater than 1.25 or less than 0.8, and the *p* value was less than 0.05 (using Student’s *t*-test). Through this screening method, 13 endogenous metabolites were identified, which might serve as potential biomarkers for differentiating the rheumatoid arthritis model group from the healthy control group (Table 2). Biomarkers were structurally identified by matching accurate mass measurements (mass error < 10 ppm) and MS/MS fragmentation patterns against the HMDB and KEGG databases. The identified biomarkers encompassed multiple metabolic classes, including amino acid derivatives (phenylacetylglycine, N-acetyl-L-glutamate, urocanate, 4-aminobutanoate, hippurate), organic acids involved in energy metabolism (citrate, 2-methylcitrate), lipid mediators (linoleate), and other metabolites (sn-glycero-3-phosphocholine, D-gluconic acid). Compared to the C group, most biomarkers were significantly reduced in the M group, with the exception of linoleate, which was markedly elevated. Mendelian randomization (MR) and meta-analysis have demonstrated that genetically predicted higher circulating linoleic acid levels are causally associated with an increased risk of inflammatory arthritic diseases, which also supports the findings of this study. Importantly, HGWD treatment effectively reversed the levels of these dysregulated metabolites towards the normal state, as visualized in the heatmap (Figure 3E), demonstrating its potent regulatory effects on RA-associated metabolic disturbances.

To systematically elucidate the metabolic pathways affected by RA and modulated by HGWD, pathway enrichment analysis was performed via MetaboAnalyst 5.0. Based on the 13 identified biomarkers, 23 metabolic pathways were significantly enriched. The most impacted pathways, ranked by *p*-values and pathway impact scores, included the metabolisms of linoleic acid, arachidonic acid, alanine–aspartate–glutamate, tryptophan, phenylalanine, arginine, and proline, as well as the tricarboxylic acid (TCA) cycle (Figure 3F). These pathways fundamentally participate in inflammatory lipid metabolism, energy homeostasis, and amino acid catabolism, all of which are known to be dysregulated in RA pathogenesis.

Importantly, the metabolic pathways identified in this study are mechanistically interconnected with the inflammatory signaling pathways predicted by network pharmacology analysis. Linoleic acid serves as a precursor for arachidonic acid, which is further metabolized by cyclooxygenases (COX) and lipoxygenases (LOX) to generate pro-inflammatory eicosanoids, including prostaglandins and leukotrienes. These lipid mediators can activate the NF-κB and MAPK cascades, subsequently upregulating the expression and release of pro-inflammatory cytokines [40]. Furthermore, dysregulation of amino acid metabolism, specifically tryptophan and arginine metabolism, has been shown to influence T-cell differentiation and function [41]. Specifically, the altered tryptophan metabolism through kynurenine pathway will affect Th17/Treg balance, and the amount of arginine will affect the proliferation of T cells and cytokine production [42,43]. In addition, the impairment of TCA circulation function may affect the energy supply required for the activation and proliferation of immune cells, thus changing the inflammatory microenvironment [44]. These results suggest that the metabolic changes observed in CIA rats are not only the consequence of inflammation, but also may directly affect the differentiation of Th17 cells and the imbalance of the TNF and IL-17 pathways. The convergence of metabolomics and network pharmacology results provides a comprehensive mechanistic framework and a solid rationale for the subsequent experimental validation of these key inflammatory pathways.

### 2.5. HGWD Inhibited TNF, Th17 Cell Differentiation, and IL-17 Signaling in CIA Rats

Based on the convergent findings from network pharmacology and metabolomics analyses, the TNF signaling, Th17 cell differentiation, and IL-17 pathways were considered the primary mechanistic targets of HGWD in RA treatment [45]. To experimentally validate these predictions, we systematically examined the key inflammatory cytokines’ levels and the stimulation status of downstream signaling molecules in CIA rats.

**Figure 4:**
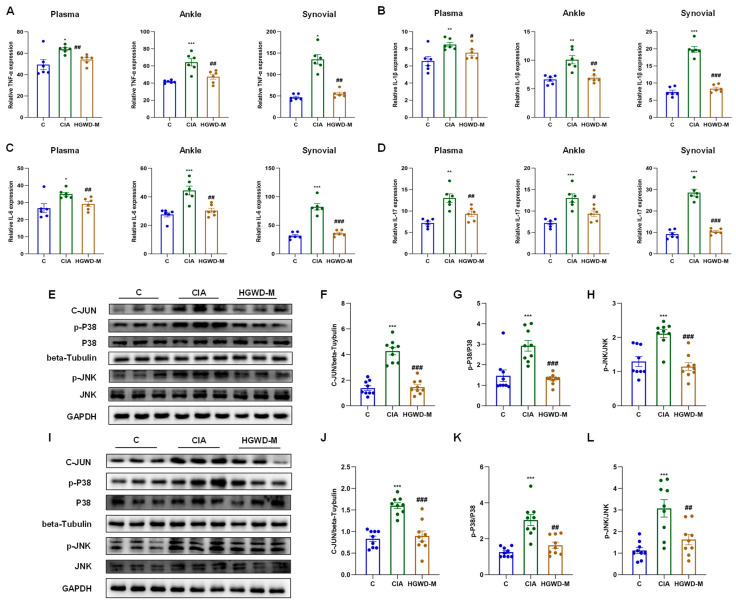
HGWD ameliorates inflammatory responses and suppresses the MAPK pathway in a rat model of arthritis. (**A**–**D**) Systemic and local pro-inflammatory cytokine profiles. Effects of HGWD on the concentrations of TNF-α (**A**), IL-1β (**B**), IL-6 (**C**), and IL-17 (**D**) in rat plasma, synovial tissues, and ankle tissues. (**E**–**H**) Protein expression and activation of MAPK pathway components in ankle tissue: representative western blots of C-JUN, p-P38, P38, p-JNK, and JNK (**E**), quantitative analysis of the relative expression of C-JUN (**F**), and the ratio of p-P38/P38 (**G**) and the ratio of p-JNK/JNK (**H**) in ankle tissue. (**I**–**L**) Protein expression and activation of MAPK pathway components in synovial tissue: representative western blots of C-JUN, p-P38, P38, p-JNK, and JNK (**I**), quantitative analysis of the relative expression of C-JUN (**J**), and the ratio of p-P38/P38 (**K**) and the ratio of p-JNK/JNK (**L**) in synovial tissue. Data are reported as mean ± SD (*n* = 3). * *p* < 0.01, ** *p* < 0.001, and *** *p* < 0.0001 vs. the blank; # *p* < 0.01, ## *p* < 0.001, and ### *p* < 0.0001 vs. the model.

Pro-inflammatory cytokines, particularly TNF-α and IL-1β/6/17, serve as critical effector molecules within the TNF and IL-17 signaling cascades and are pivotal drivers of Th17 cell-mediated inflammatory responses. As illustrated in Figure 4A–D, the Model group exhibited markedly elevated concentrations of TNF-α (Figure 4A) and IL-1β/6/17 (Figure 4B–D) in plasma, synovial tissues, and ankle tissues compared to the Control (*p* < 0.05), validating the effective establishment of the inflammatory milieu characteristic of RA. It is worth noting that HGWD could reduce the over expression of the above inflammatory mediators in a dose-dependent manner (*p* < 0.05), and its curative effect was equivalent to that of the positive control MTX, reflecting its significant anti-inflammatory ability. In order to further clarify the molecular mechanism of anti-inflammatory effect of HGWD, this study explored the activation of the MAPK signaling cascade, which is a key downstream effector link of TNF and IL-17 signaling and is involved in the regulation of cytokine production and the inflammatory response [39,46]. The results of western blotting showed that compared with the control group, CIA induction significantly increased the expression and phosphorylation level of key MAPK components (including P38, p-P38, JNK, and p-JNK) and transcription factor C-JUN in ankle joint and synovial tissue (*p* < 0.05) (Figure 4E–L). Importantly, HGWD treatment significantly reversed these aberrant activations (*p* < 0.05), indicating its capacity to suppress MAPK-mediated inflammatory signaling. Given that the MAPK pathway serves as a convergent node linking TNF-α and IL-17 receptor signaling to downstream transcriptional responses, including AP-1 activation and subsequent cytokine gene expression, these outcomes present mechanistic evidence that HGWD has its anti-arthritic effects by interrupting the positive feedback loop between pro-inflammatory cytokines and MAPK activation [39,47,48].

Collectively, these results validated that HGWD ameliorates RA progression through coordinated inhibition of Th17 cell differentiation and the TNF and IL-17 pathways, corroborating the predictions derived from network pharmacology analysis. Having established the in vivo efficacy and mechanistic basis of HGWD, we next sought to validate the biological activity of its key active constituents in cellular models.

### 2.6. Anti-Inflammatory Effects of the Key Active Compound Combination from HGWD Validated in Cellular Models

To further substantiate the pharmacological basis of HGWD identified through network pharmacology analysis, we evaluated the anti-inflammatory activity of both the whole HGWD extract and the active compound combination (ACC) in cellular models. Based on the network pharmacology predictions and topological analysis, four compounds—calycosin, paeoniflorin, 6-gingerol, and formononetin—were selected as the representative active constituents of HGWD due to their high degree values in the CTP network and their well-documented anti-inflammatory properties. These four compounds were combined in proportions reflective of their relative abundance in the HGWD decoction to constitute the ACC for subsequent validation studies (the mass ratio of paeoniflorin, calycosin, formononetin, and 6-gingerol is 296:6:1:17) Two complementary cellular models were employed to recapitulate distinct aspects of RA pathogenesis: LPS-stimulated RAW264.7 murine macrophages, which model the innate immune inflammatory response [49], and TNF-α-induced MH7A human rheumatoid arthritis synovial fibroblasts, which represent the pathological behavior of activated synoviocytes [48]. The anti-inflammatory effect is evaluated by measuring the production of nitric oxide (NO), the secretion of pro-inflammatory cytokines, and the ability of cell migration and invasion.

**Figure 5:**
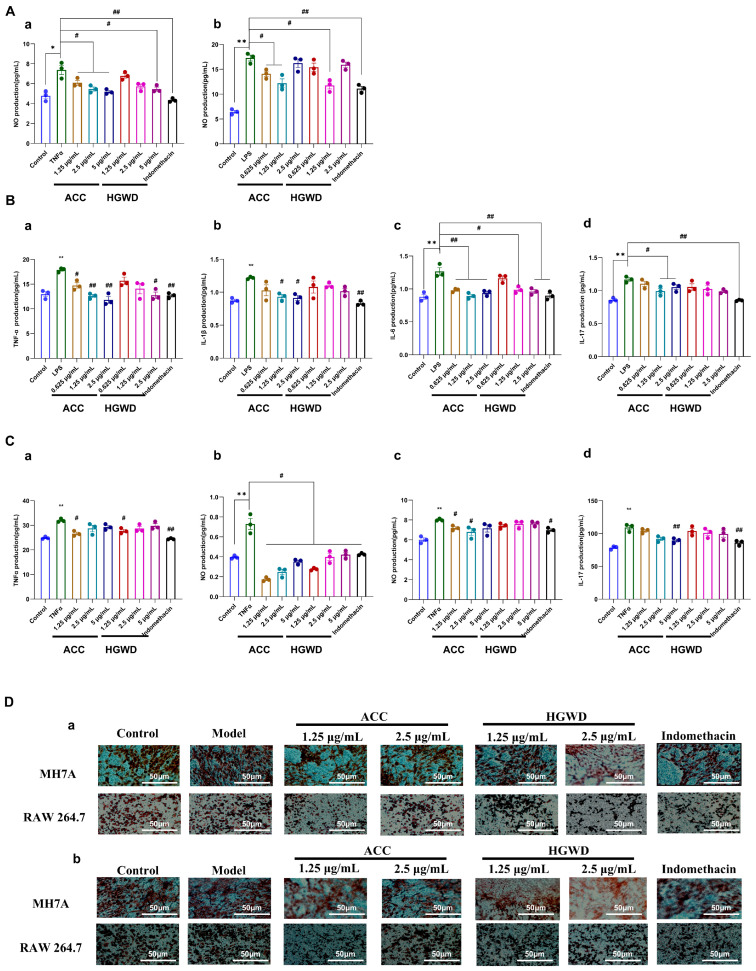
HGWD and ACC attenuate inflammatory responses and pathological behaviors in RA cell models. (**A**) Effects of ACC and HGWD on NO production in (**a**) MH7A and (**b**) RAW264.7 cells. (**B**) Effects of HGWD and ACC on TNF-α (**a**), IL-1β (**b**), IL-6 (**c**), and IL-17 (**d**) levels in RAW264.7 cells. (**C**) Effects of HGWD and ACC on the levels of TNF-α (**a**), IL-1β (**b**), IL-6 (**c**), and IL-17 (**d**) in MH7A cells. (**D**) Effects of HGWD and ACC on migration (**a**) and invasion (**b**) of MH7A and RAW264.7 cells. “*” *p* < 0.05 or “**” *p* < 0.01 vs. control; “#”, *p* < 0.05 or “##” *p* < 0.01 vs. the TNF-α group.

As shown in Figure 5A, LPS stimulation significantly increased the production of NO in RAW264.7 cells, while 1.25 µg/mL HGWD extract significantly inhibited this effect. The active chemical compounds (ACC) showed the same or even stronger inhibitory effect as HGWD, indicating that the combination of purified active ingredients had better inhibitory effect. Similarly, in MH7A cells induced by TNF-α, 5 µg/mL HGWD extraction can effectively reduce the production of NO, while ACC can achieve similar effects at the concentrations of 1.25, 2.5 and 5 µg/mL, suggesting that ACC has stronger anti-inflammatory activity at the same concentration. Consistent with the NO inhibition results, ELISA analysis showed that HGWD and ACC could significantly inhibit the release of pro-inflammatory cytokines in RAW264.7 (Figure 5B) and MH7A cells (Figure 5C). It is worth noting that at the same concentration, the inhibitory effect of ACC on cytokine production is significantly stronger than that of HGWD, which further supports that the four compound combination is the main material basis for the anti-inflammatory effect of HGWD. In addition to regulating the pathological migration and invasion of cytokines, inflammatory cells, and synovial fibroblasts, it is also the key cause of pannus formation and joint destruction in RA [50,51]. The transwell migration assay showed that HGWD and ACC significantly inhibited the chemotaxis of LPS-stimulated RAW264.7 macrophages and TNF-α-induced MH7A synovial fibroblasts (Figure 5D-a). Furthermore, the matrix gel invasion assay demonstrated that both treatments significantly reduced the invasion ability of the aforementioned cells (Figure 5D-b). These results indicate that HGWD and its effective components can not only inhibit the production of inflammatory mediators, but also inhibit the aggressive cell behaviors that lead to synovial hyperplasia and cartilage erosion in RA.

Collectively, these in vitro findings validated that the four key active compounds—calycosin, paeoniflorin, 6-gingerol, and formononetin—recapitulate and potentially enhance the anti-inflammatory and anti-invasive effects of the parent HGWD formula.

### 2.7. Pharmacokinetic Characteristics of the Active Compounds in HGWD

Having demonstrated the in vitro anti-inflammatory effectiveness of the active compound combination (ACC), we next investigated the pharmacokinetic profiles of these four key constituents to assess their in vivo exposure and bioavailability following oral administration of HGWD. Understanding the pharmacokinetic behavior of these compounds is essential for establishing the correlation between systemic exposure and therapeutic efficacy, thereby providing a rational basis for their identification as the pharmacodynamic material basis of HGWD.

**Figure 6:**
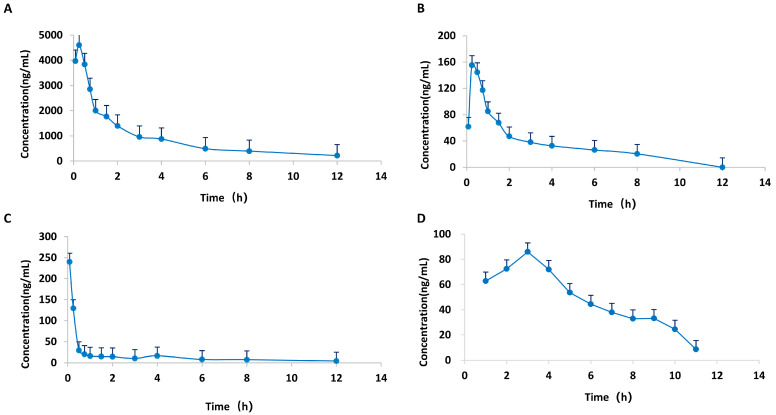
Mean plasma concentration–time curves of four active compounds from HGWD in plasma from CIA rats. (**A**) paeoniflorin; (**B**) calycosin; (**C**) 6-Gingerol; and (**D**) formononetin.

A HPLC-MS/MS technique was employed for the quantification of the plasma levels of calycosin, paeoniflorin, 6-gingerol, and formononetin in CIA rats following oral administration of HGWD at 15 g/kg/day. Pharmacokinetic parameters were measured via a non-compartmental model, and Table 3 summarizes the outcomes. The plasma concentration–time profiles over a 24 h period are illustrated in Figure 6. The four compounds showed good oral absorption characteristics, and reached the plasma peak concentration (Tmax) in a short time. Among them, the absorption of 6-gingerol was the fastest, with a Tmax of 0.23 h, followed by paeoniflorin (0.28 h), calycosin (0.31 h) and formononetin (0.89 h). This rapid absorption is conducive to the rapid reaching of effective therapeutic concentration of the drug after taking.

Among the four active compounds, paeoniflorin has the best pharmacokinetic performance, and its maximum plasma concentration (C_max_ = 5143.50 ± 2302.67 mg/L) and the area under the concentration–time curve (AUC_0–24h_ = 10,137.39 ± 1825.85 mg/L H) are the highest, which were significantly higher than the other three compounds. This indicates that paeoniflorin is the most important active component of HGWD in the blood. The high systematic exposure of paeoniflorin is consistent with its core hub position in network pharmacological analysis, which further confirms the important contribution of paeoniflorin to the overall efficacy of HGWD.

In comparison, calycosin (C_max_ = 201.63 ± 89.40 mg/L; AUC_0–24h_ = 352.40 ± 17.01 mg/L·h), 6-gingerol (C_max_ = 131.54 ± 183.82 mg/L; AUC_0–24h_ = 142.11 ± 35.72 mg/L·h), and formononetin (C_max_ = 95.60 ± 36.14 mg/L; AUC_0–24h_ = 267.79 ± 53.79 mg/L·h) displayed moderate but measurable plasma concentrations, indicating their bioavailability and potential contribution to the pharmacological effects.

Pharmacokinetic analysis confirmed that after oral administration of HGWD, four key active components—calycosin, paeoniflorin, 6-gingerol and formononetin—could be absorbed into the blood circulation and reached detectable plasma concentrations. This was consistent with their observed pharmacological activities. The favorable absorption characteristics and adequate systemic exposure of these constituents provide pharmacokinetic evidence supporting their identification as the principal bioactive components responsible for the anti-arthritic effects of HGWD. Based on these results, together with the in vivo effect in CIA rats and the in vitro verification in cell models, this study provides a comprehensive pharmacological basis for the therapeutic principle of HGWD in the treatment of RA.

## 3. Discussion

As a complex immune system disease, the treatment of RA is often difficult to achieve ideal results through intervention at a single target, and multi-target collaborative intervention may provide a more comprehensive treatment strategy [52]. TCM formulations, distinguished by their multi-component nature, offer a promising reservoir of natural products capable of simultaneously modulating diverse pathological pathways [53,54,55]. By integrating serum pharmacochemistry, metabolomics, network pharmacology, and experimental verification, this study systematically clarified the pharmacodynamic material basis and multi-target mechanism of HGWD in the treatment of RA. Thus, from the perspective of coordinating the regulation of inflammatory signals and metabolic homeostasis, it revealed the therapeutic principle of this classic compound.

The modernization research of TCM faces a core scientific challenge, which is how to accurately identify and confirm the bioactive components that really play a therapeutic role from its complex chemical system [56,57]. Traditional phytochemical methods aim to comprehensively characterize all compounds in herbal extracts, but it is difficult to distinguish pharmacologically active components from concomitant ineffective components. Serum pharmacochemistry effectively overcomes this limitation by focusing on compounds that enter the systemic circulation after oral administration, because these “blood migration components” are more likely to reach the target and produce therapeutic effects [23,58]. In this study, 99 chemical components were identified from HGWD by liquid chromatography tandem mass spectrometry, of which 25 prototype compounds could be detected in rat plasma. These absorbed components come from all five herbs, including flavonoids, monoterpenoids, gingerols, and phenylpropanoids. It is worth noting that there are several of these ingredients, such as calycosin, formononetin, paeoniflorin, and 6-gingerol, that were already known to be anti-inflammatory and immune-regulating. This also provides a reasonable chemical explanation for the efficacy observed by HGWD.

The analysis of network pharmacology shows that the components absorbed by HGWD will act on several key inflammatory and immunomodulatory pathways closely related to RA. Among them, TNF signaling, IL-17 signaling, and the Th17 cell differentiation pathway are the main targets, which is in line with the known role of these pathways in driving synovitis, pannus formation, and bone destruction in RA [59,60,61]. Th17 cells, as a subset of CD4 + helper T cells, secrete high levels of IL-17 and cooperate with other proinflammatory factors (such as TNF-α, IL-1 β, IL-6) to jointly drive the characteristic inflammatory cascade and tissue destruction process in RA [62,63,64,65,66]. These cytokines stimulate synovial fibroblasts and resident macrophages to produce additional inflammatory mediators, creating a self-amplifying inflammatory loop. Moreover, IL-17 and TNF-α can directly activate endothelial cells and make macrophages secrete VEGF to promote angiogenesis, thus maintaining the formation of pannus [67,68,69]. Its function of destroying bone is mainly realized through IL-17 upregulating the ligand of nuclear factor kappa B receptor activating factor, which can promote osteoclastogenesis and inhibit chondrocyte function [70,71,72]. Experiments in CIA rats verified these predictions, and it was proved that HGWD could significantly block the production of TNF-α and IL1β/6/17 and inhibit the phosphorylation of key MAPK pathway components, including P38 and JNK, thus interrupting the positive feedback cycle of cytokine–MAPK that keeps inflammation going.

In addition to the research results of network pharmacology, non-targeted metabolomics analysis shows that HGWD treatment can significantly improve metabolic disorders in the CIA rat model. We found 13 potential biomarkers, including amino acid derivatives, organic acids, and lipid mediators, etc. These metabolites mainly involved key metabolic pathways such as linoleic acid metabolism, arachidonic acid metabolism, amino acid metabolism, and the TCA cycle. These metabolic pathways are closely related in mechanism to the inflammatory signaling cascade identified through network pharmacology. Specifically, as a precursor of arachidonic acid, linoleic acid produces pro-inflammatory eicosanoids (such as prostaglandins, leukotrienes, etc.) that can further activate key inflammatory signaling pathways such as NF-κB and MAPK, thereby forming a positive feedback loop between metabolic disturbances and inflammation amplification [73,74,75]. The disorder of tryptophan and arginine metabolism can affect the differentiation and function of T cells, especially the regulation of Th17/Treg cell balance [76,77]. The integration of metabolomics and network pharmacology results suggests that the metabolic abnormalities observed in the CIA model are not only a consequence of inflammation, but also likely to actively drive immune dysregulation. HGWD exerts its therapeutic effect by synergistically regulating inflammatory signals and metabolic homeostasis. It should be noted that the non-targeted metabolomics employed in this study is a research strategy without pre-set hypotheses, aimed at comprehensively characterizing the overall metabolic changes caused by HGWD intervention. Although this method is particularly suitable for the initial discovery of new metabolic characteristics and pathways, it has inherent limitations in quantitative accuracy compared with targeted metabonomics. In the future, it is necessary to use targeted metabonomics technology quantitatively verify the key differential expressed metabolites identified in this study and further clarify their mechanism in the therapeutic effect of HGWD.

Through systematic identification and multi-dimensional validation, this study successfully established a four-component active combination consisting of calycosin, paeoniflorin, 6-gingerol, and formononetin. This combination can be regarded as the representative pharmacological material basis of HGWD. These four compounds were selected based on their central hub position in the “component-target-pathway” (CTP) network (with the highest degree among 12 active compounds) and were further verified through a comprehensive experimental system that encompassed in vitro cell models, in vivo CIA efficacy evaluation, and pharmacokinetic analysis. This active combination exhibits anti-inflammatory and anti-migratory activities comparable or superior to the HGWD full extract in vitro. It effectively inhibited NO production and pro-inflammatory cytokine secretion, and significantly inhibited the migration and invasion of macrophages and synovial fibroblasts. Pharmacokinetic studies have confirmed that all four compounds can reach detectable plasma concentrations after oral administration, thereby establishing a crucial “absorption–exposure” correlation. This verifies that they are genuine bioactive constituents with bioavailability, rather than merely compounds with theoretical predictive potential.

The key is that pharmacokinetic analysis provides crucial evidence of the “absorption–exposure–effect” correlation, which not only verifies the bioactive ingredients predicted by network pharmacology, but also connects the prediction in vitro with the curative effect in vivo. Drug discovery guided by network pharmacology follows a fundamental premise: the predicted active ingredients must reach a systemic exposure level sufficient to activate their targets in vivo [78]. However, in the mechanism research of TCM, this pharmacokinetic verification is often ignored. In this study, we found that the four key components—calycosin, paeoniflorin, 6-gingerol and formononetin—were absorbed quickly by oral administration, and the T_max_ value was between 0.23 and 0.89 h, indicating that the therapeutic concentration could be reached quickly after oral administration of HGWD. This rapid absorption has pharmacological advantages for inflammatory diseases that need timely intervention.

Critically, the pharmacokinetic characteristics are highly consistent with the network pharmacological prediction and topology analysis. paeoniflorin exhibits the highest systemic exposure, which was consistent with its highest connectivity in the CTP network and its extensive interactions with multiple inflammatory targets including TNF-alpha, IL-6, and key components of the MAPK signaling pathway. This favorable pharmacokinetic profile provides a quantitative basis for understanding paeoniflorin’s predominant contribution to the overall anti-inflammatory effects of HGWD observed in the CIA model. In comparison, the plasma concentrations of calycosin, 6-gingerol, and formononetin were moderate but undetectable, and their unique pharmacokinetic behavior suggested that the combination might play its role through a time coordinated multi-target regulation mode rather than a simple synchronous effect. This sequential exposure mode, in which each component reaches its peak concentration at different times, may form the basis for the sustained efficacy of the compound and realize the continuous regulation of the relevant pathways during the whole dosing interval.

An important finding of this study is that the four compounds exhibiting the highest centrality scores in network pharmacology and experimentally confirmed anti-inflammatory activity were derived from just three of five component herbs: *Astragalus membranaceus* (calycosin, formononetin), *Paeoniae radix* Alba (paeoniflorin), and *Zingiberis rhizoma* (6-gingerol). Although several absorbed compounds were identified from *Cinnamomi ramulus* and *Jujubae fructus* (Table 1), none appeared as central bioactive agents in the network analysis or within the validated active combination. However, this outcome requires careful interpretation due to methodological and theoretical factors. Serum pharmacochemistry primarily detects absorbed parent compounds, potentially overlooking substances with local gastrointestinal activity, bioactive metabolites formed post-metabolism, compounds present at undetectable yet active concentrations, or synergistic interactions that enhance the efficacy of other components [79,80]. Furthermore, according to traditional Chinese medicine principles, *Cinnamomi ramulus* (Guizhi) and *Jujubae fructus* (Dazao) may contribute through functions like promoting circulation, warming channels, harmonizing the formula, and supporting spleen health—effects not directly measured by anti-inflammatory cytokine assays but potentially influencing therapeutic outcomes via metabolic or gut-mediated mechanisms [81]. Nevertheless, the study indicates that the anti-rheumatoid arthritis activity of HGWD can be largely represented by a simplified combination of four compounds from three herbs, offering valuable guidance for formula optimization and standardization. Future investigations should employ comprehensive metabolomics to identify active metabolites, analyze gut microbiome interactions, and compare the efficacy of the full formula with reduced herb or compound combinations to clarify the role of each constituent.

**Figure 7:**
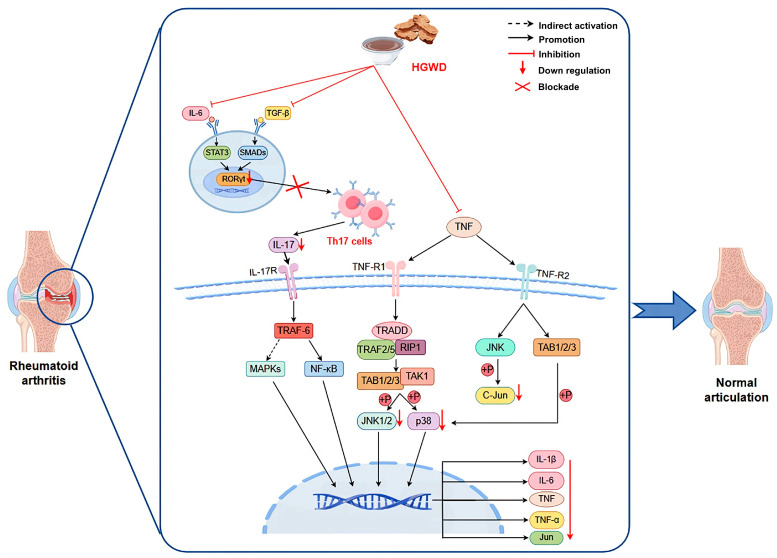
HGWD interferes with RA based on TNF, Th17 cell differentiation, and IL-17 pathways.

This study systematically illustrates how HGWD, as a multi-component TCM formula, exerts anti-arthritis effects by synergistically regulating inflammatory pathways and metabolic homeostasis (Figure 7). The integration strategy adopted covers serum pharmacochemistry, metabonomics, network pharmacology and experimental verification, providing an efficient research paradigm for the study of multi-target natural products. We confirmed that calycosin, paeoniflorin, 6-gingerol, and formononetin were the main active ingredients, which not only explained why HGWD was effective, but also provided lead compounds for developing new multi-target drugs to treat RA (rheumatoid arthritis) and even other inflammatory diseases. These findings contribute to the broader understanding of multi-targeted natural products as therapeutics and support the continued exploration of TCM formulations as sources of innovative drug candidates.

## 4. Materials and Methods

### 4.1. Chemicals and Reagents

Reference standards, such as calycosin, paeoniflorin, albiflorin, 6-gingerol, formononetin, and cinnamic acid (purity > 98%), were obtained from Weikeqi Biotechnology Co., Ltd. (Chengdu, China). MTX was brought from Pfizer Pharmaceuticals Ltd. (Beijing, China) and used as a positive control. ELISA kits for IL-1β/6/17 and TNF-α were brought from Shanghai Luyu Biotechnology Co., Ltd. (Shanghai, China). Bovine type II collagen (CII) and complete Freund’s adjuvant (CFA) were from Chondrex Inc. (Redmond, WA, USA). HPLC-grade methanol and acetonitrile were obtained from Thermo Fisher Scientific (Waltham, MA, USA). Finally, the preparation of ultrapure water was conducted via a Milli-Q Integral system (Millipore, Burlington, MA, USA). Other chemicals were of analytical grade.

### 4.2. Herbal Materials and Preparation of the HGWD Extract

The five medicinal herbs constituting HGWD, namely *Astragali radix* (Huangqi), *Cinnamomi ramulus* (Guizhi), *Paeoniae radix* Alba (Baishao), *Zingiberis rhizoma* Recens (Shengjiang), and *Jujubae fructus* (Dazao), were brought from Hebei Renxin TCM Co., Ltd. (Chengdu, China). All herbal materials were validated by Professor Wang Dong at the School of TCM, Shenyang Pharmaceutical University. We prepared the HGWD extract as per the traditional decoction method. Briefly, the five herbs were combined in the classical ratio (*Astragali radix*/*Cinnamomi ramulus*/*Paeoniae radix* Alba/*Zingiberis rhizoma* Recens/*Jujubae fructus* = 3:3:3:6:4, *w*/*w*), soaked in 10 volumes of distilled water for 30 min, and decocted twice (1 h each). We concentrated the combined filtrates under lowered pressure via a rotary evaporator and subsequently lyophilized to obtain the HGWD extract powder, which was stored at −20 °C until use.

### 4.3. CIA Model Establishment and Drug Administration

Male Sprague-Dawley (SD) rats (180–220 g, 6–8 weeks old) were obtained from the Animal Experimental Center (Shenyang, China; Certificate No. SCXK2020-0001) and kept under specific pathogen-free (SPF) environment with regulated temperature (22 ± 2 °C), humidity (55 ± 5%), and a 12 h light/dark cycle. Rats had unrestricted standard food and water. After a 7-day acclimatization, animals were classified into experimental groups in a random manner. All procedures received approval from the Ethics Committee of Animal Experiments at the General Hospital of Northern Theater Command (No. 2023-012) and were conducted as per the NIH Guide for the Care and Use of Laboratory Animals.

The CIA model was established as previously mentioned with minor modifications [48]. After dissolving Bovine CII in 0.1 M acetic acid (2 mg/mL) overnight at 4 °C, emulsification was conducted with an equivalent volume of CFA to 1 mg/mL. On day 0, rats received 0.3 mL of the CII/CFA emulsion subcutaneously at two sites on the back and one site at the tail base. A booster injection was administered on day 14 at the same dose and sites. An equal volume of normal saline was administered to control rats. On day 21, rats with an arthritis score ≥ 4 were selected and classified into six groups (*n* = 8/group) in a random manner: (i) Control group (normal rats, saline); (ii) Model group (CIA rats, saline); (iii) MTX group (CIA rats, 3 mg/kg MTX, twice weekly); (iv) HGWD-L group (CIA rats, 7.5 g/kg/day HGWD); (v) HGWD-M group (CIA rats, 15.0 g/kg/day HGWD); and (vi) HGWD-H group (CIA rats, 30.0 g/kg/day HGWD). An additional eight CIA rats in the HGWD-M group were designated for serum pharmacochemistry analysis. All treatments were received intragastrically once daily for 28 consecutive days. Paw volume, body weight, and arthritis scores were monitored every 3 days throughout the treatment period.

### 4.4. Assessment of Arthritis Severity

#### 4.4.1. Paw Volume and Arthritis Index Measurements

Hind paw volumes were assessed via a PV-200 plethysmometer (TECHMAN Soft, Chengdu, China) at 3-day intervals during the treatment period. Paw swelling rate was calculated as follows: (Vt − V0)/V0 × 100%, where Vt denotes the paw volume at time t and V0 denotes the initial paw volume. Arthritis severity was assessed via a semi-quantitative scoring system: 0, normal; 1, localized swelling and erythema confined to toe joints; 2, swelling involving both toe joints and dorsum pedis; 3, entire paw affected with moderate erythema; 4, severe swelling and erythema throughout the paw with motor dysfunction. The arthritis index for each rat was calculated as the sum of scores from both hind paws (maximum score = 8).

#### 4.4.2. Histopathological Examination

Ankle tissues were embedded in paraffin following decalcification as previously described [48]. Paraffin blocks were baked for 4 h, and sections were cut, baked for 30 min, dewaxed with xylene, and washed with ethanol and PBS. Slices underwent hematoxylin and eosin, staining, dehydrating, clearing, mounting, and imaging via a microscope (BC46, Olympus, Tokyo, Japan) at 200× magnification.

### 4.5. Sample Preparation

Collecting blood samples from the orbital sinus was conducted under isoflurane anesthesia. For serum pharmacochemistry analysis, blood samples from HGWD-M group rats were collected at 1, 2, 4, and 6 h post-administration. For pharmacodynamic evaluation, the collection of blood samples was conducted at 12 h post-administration from all groups. Blood was immediately anticoagulated with heparin sodium and spun at 3000× *g* for 10 min at 4 °C. Plasma separation was conducted and maintained at −80 °C until analysis. At the study endpoint, rats were euthanized by intraperitoneal injection of pentobarbital sodium (45 mg/kg), and ankle joint tissues were collected for histopathological examination.

### 4.6. Serum Pharmacochemistry Analysis

#### 4.6.1. Plasma Sample Preparation

Blank plasma samples (pre-dose) from the six rats were pooled, and post-dose plasma samples were independently pooled for each time point. Protein precipitation was conducted by applying 300 μL of ice-cold methanol to 100 μL of pooled plasma, and then vortexing was conducted for 3 min and spinning at 10,142× *g* was conducted for 5 min at 4 °C. The transfer of supernatant was conducted to a clean tube and evaporation was conducted to dryness under nitrogen at 37 °C. The residue underwent reconstitution in 100 μL of 75% methanol (*v*/*v*), vortexing, ultrasonication for 5 min, and centrifuging at 10,142× *g* for 5 min. The resulting supernatant was transferred to an autosampler vial for HPLC-Q-TOF-MS/MS analysis.

#### 4.6.2. HPLC-Q-TOF-MS/MS Conditions

Chromatographic separation was conducted on an Agilent 1290 Infinity II HPLC system (Santa Clara, CA, USA) with a ZORBAX Eclipse Plus C18 column (2.1 × 100 mm, 1.8 μm, Agilent Technologies, Santa Clara, CA, USA). Supplementary Materials present detailed descriptions.

### 4.7. Network Pharmacology Analysis

Plasma prototype components identified from component analysis were chosen for target investigation. Component targets were predicted via SwissTargetPrediction (http://www.swisstargetprediction.ch/, opened 24 February 2023), HERB (http://herb.ac.cn/, opened 20 March 2023), and SEA (https://sea.bkslab.org/, opened 20 March 2023). For the SWISS and SEA databases, a screening threshold of prediction score > 0.5 was applied. Disease targets for “Rheumatoid arthritis” were obtained from GEO (https://www.ncbi.nlm.nih.gov/geo/ opened 20 March 2023) and GeneCards (https://www.genecards.org/, opened 20 March 2023). For the GEO database, genes were screened using the criteria of *p*-adj < 0.05 and logFC > 0. For the GeneCards database, targets with relevance scores greater than twice the average value were selected. Overlapping component and disease targets were utilized to create a PPI network via STRING (https://cn.string-db.org/, accessed 26 January 2023). KEGG analysis of key targets was conducted via Bioinformatics (https://academic.oup.com/bioinformatics/, accessed 28 January 2023), and Cytoscape 3.9.0 (San Francisco, CA, USA) was utilized to observe the component–target–pathway network.

### 4.8. ELISA

Serum levels of inflammatory cytokines and autoimmune markers (anti-CII antibody, rheumatoid factor, and C-reactive protein) were assessed via commercial ELISA kits as per the manufacturers’ protocols. A 100 μL of diluted serum or standards was applied to pre-coated plates of 96 wells and incubation was conducted at 37 °C for 90 min. After washing, biotinylated detection antibodies were added for 60 min, and then streptavidin-HRP for 30 min. Color development was conducted with TMB substrate and blocked with the provided solution. Absorbance was assessed at 450 nm (Multiskan FC, Thermo Fisher Scientific, USA), and levels were determined from standard curves.

### 4.9. Western Blotting Analysis

Total protein isolation from ankle joint tissues was conducted via RIPA buffer with protease and phosphatase inhibitors. Protein levels were assessed with a BCA assay kit (P0010S, Beyotime, Shanghai, China). The separation of equal protein amounts (10 μg/lane) was conducted by 10% SDS-PAGE, followed by transferring to PVDF membranes. The blockage of membranes was conducted with 5% non-fat milk in TBST for 1.5 h at room temperature, and overnight incubation was conducted at 4 °C with primary antibodies: anti-c-Jun (Proteintech, Rosemont, IL, USA), anti-p38 (Proteintech), anti-phospho-p38 (Cell Signaling Technology, Danvers, MA, USA), anti-JNK (Proteintech), anti-phospho-JNK (Cell Signaling Technology), and anti-β-tubulin (Proteintech) (All 1:1000, β-tubulin 1:5000). After rinsing with TBST, a 1 h incubation of membranes was conducted with HRP-conjugated secondary antibodies (1:5000, Proteintech) at room temperature. Bands were observed via enhanced chemiluminescence, and ImageJ (v1.52, NIH, Bethesda, MD, USA) was utilized for intensity quantification, normalized to β-tubulin.

### 4.10. Untargeted Metabolomics Analysis

A global non-targeted metabolomic analysis was performed on plasma samples following our previously established protocol [82]. Detailed procedures for sample preparation, mass spectrometry, liquid chromatography, and data processing are provided in the Supplementary Materials.

### 4.11. Cell Culture and Treatment

Murine macrophage RAW264.7 cells and human synovial fibroblast MH7A cells were acquired from the American Type Culture Collection (ATCC, Manassas, VA, USA) and cultivated in Dulbecco’s modified Eagle’s medium (DMEM) enriched with 100 U/mL penicillin, 10% fetal bovine serum (FBS), and 100 μg/mL streptomycin at 37 °C in humidified conditions with 5% CO_2_. The active compound combination (ACC), comprising calycosin, paeoniflorin, 6-gingerol, and formononetin, was dissolved in dimethyl sulfoxide (DMSO) to a stock level of 10 mg/mL, with the final DMSO level kept < 0.1% (*v*/*v*).

RAW264.7 cells were plated in 96-well plates (4.0 × 10^3^ cells/well) and maintained overnight. Cells were pretreated with ACC (0.625, 1.25, and 2.5 μg/mL) for 24 h and subsequently stimulated with lipopolysaccharide (LPS, 1 μg/mL) for 12 h. Cell viability was assessed by a MTT assay to confirm non-cytotoxic conditions. NO generation in supernatants was quantified via the Griess reagent assay. We incubated a mixture of 100 μL of supernatant and an equivalent volume of Griess reagent (1% sulfanilamide in 5% phosphoric acid and 0.1% N-(1-naphthyl) ethylenediamine dihydrochloride) at room temperature for 10 min, after which absorbance was read at 540 nm. NO levels were assessed via a sodium nitrite standard curve (0–100 μM).

### 4.12. Transwell Migration Assay

Cell migration was estimated via Transwell chambers (8 μm pore size, Corning, NY, USA). For migration assays, a suspension of MH7A cells (7 × 10^4^ cells) in 100 μL of serum-free medium was seeded into the top chamber, while 600 μL of complete medium with 10% FBS was applied to the bottom chamber. For invasion assays, we pre-coated the top chamber with Matrigel matrix (Corning, NY, USA) diluted 1:8 in serum-free medium at 37 °C for 2 h before cell seeding. After incubation for 24 h at 37 °C, non-migrated cells on the top membrane surface were eliminated with cotton swabs. A 4% paraformaldehyde solution was utilized to fix cells that transferred to the bottom surface for 30 min, and 1% crystal violet was utilized to stain them for 3 min. We counted migrated cells in five random fields/chamber under a light microscope at 200× magnification.

### 4.13. Pharmacokinetic Study

The pharmacokinetic profiles of the four active compounds (calycosin, paeoniflorin, 6-gingerol, and formononetin) were characterized following oral administration of HGWD (15 g/kg) to SD rats. The collection of blood samples (~200 μL) was conducted from the orbital sinus at predefined time points (0.25, 0.5, 1, 2, 4, 6, 8, 12, and 24 h post-administration). Plasma was acquired by centrifugation and kept at −80 °C until analysis. To prepare the sample, 100 μL of plasma was combined with 10 μL of internal standard solution (10 μg/mL geniposide and 5 μg/mL 4′-O-β-glucopyranosyl-5-O-methylvisamminol) and 300 μL of methanol. After vortexing (3 min) and centrifugation (10,142× *g*, 5 min), the supernatant was evaporated to dryness under nitrogen. The residue was reconstituted in 50 μL of methanol, vortexed, ultrasonicated (5 min), and centrifuged, and the supernatant was analyzed using a validated HPLC-MS/MS technique. Pharmacokinetic parameters (C_max_, t_1/2_, AUC_0–t_, T_max_, and AUC_0–∞_) were assessed by non-compartmental analysis via DAS 2.0 (Mathematical Pharmacology Professional Committee of China). Supplementary Materials present detailed analytical conditions.

### 4.14. Statistical Analysis

Data are expressed as mean ± standard deviation (SD) from at least three independent trials. SPSS 26.0 (IBM Corp., Armonk, NY, USA) was employed for statistical analyses. Data distribution was examined using the Shapiro–Wilk test for normality, and homogeneity of variances was evaluated using Levene’s test. One-way analysis of variance (ANOVA) and then Tukey’s post hoc test were applied for multiple-group comparisons, while Student’s *t*-test was applied for two-group comparisons. *p* < 0.05 was regarded as significant and <0.01 highly significant. GraphPad Prism 9.0 (GraphPad Software, San Diego, CA, USA) was utilized for data visualization.

## 5. Conclusions

This study systematically investigated HGWD through integrated serum pharmacochemistry, metabolomics, network pharmacology, and experimental validation. A total of 99 chemical components in HGWD were identified, four key compounds—calycosin and formononetin from *Astragalus membranaceus*, paeoniflorin from *Paeoniae radix* Alba, and 6-gingerol from *Zingiberis rhizoma*—emerged as the principal bioactive constituents responsible for the anti-arthritic effects through coordinated modulation of TNF, IL-17, and Th17 cell differentiation pathways. Notably, while *Cinnamomi ramulus* and *Jujubae fructus* contributed absorbed compounds, non were identified as core therapeutic components in our analytical framework, suggesting that the primary anti-inflammatory effects may be attributable to three of the five constituent herbs. However, this does not preclude potential contributions through metabolic modulation, synergistic enhancement, or mechanisms not captured by our current methodologies. These findings provide a scientific foundation for formula optimization and the development of multi-targeted natural product-based RA therapeutics, while highlighting the need for comprehensive studies to fully elucidate the role of each constituent herb.

## Figures and Tables

**Table 1:** Identification of the chemical constituents of HGWD in a plasma sample.

No.	Identification	Formula	Adduct	*t*_R_ (min)	Calculated (*m*/*z*)	Classification
1	Choline [24] (C)	C_5_H_13_NO	[M + H]^+^	3.07	103.09971	Alkaloids
2	L(+)-Arginine [25] (A, C, D, E)	C_6_H_14_N_4_O_2_	[M + H]^+^	3.09	174.11168	Amino acids
3	Betaine [26] (A)	C_5_H_11_NO_2_	[M + H]^+^	3.12	117.07898	Alkaloids
4	DL-Proline [25] (A, D, E)	C_5_H_9_NO_2_	[M + H]^+^	3.13	115.06333	Amino acids
5	Tyrosine [24] (A, B, C, D, E)	C_9_H_11_NO_3_	[M + H]^+^	4.34	181.07389	Amino acids
6	Nicotinamide [26] (E)	C_6_H_6_N_2_O	[M + H]^+^	4.40	122.04801	Alkaloids
7	Phenylalanine [27] (A)	C_9_H_11_NO_2_	[M + H]^+^	5.51	165.07898	Amino acids
8	Luotonin [25] (A, D, E)	C_11_H_12_N_2_O_2_	[M + H]^+^	9.29	204.08988	Amino acids
9	Lactinolide [26] (C)	C_10_H_16_O_4_	[M + H]^+^	9.54	200.10486	Monoterpenoids
10	Protocatechuic acid [25] (A, C, D, E)	C_7_H_6_O_4_	[M − H]^−^	20.62	154.02661	Organic acids
11	Albiflorin [28] (C)	C_23_H_28_O_11_	[M + H]^+^	21.89	480.16316	Monoterpene glycosides
12	Albiflorin C [29] (C)	C_17_H_18_O_6_	[M + H]^+^	21.91	318.11034	Monoterpene glycosides
13	Paeoniflorin [30] (C)	C_23_H_28_O_11_	[M − H]^−^	23.49	480.16316	Monoterpene glycosides
14	Mudanpioside E [24] (C)	C_24_H_30_O_13_	[M − H]^−^	23.49	526.16864	Monoterpene glycosides
15	Azelaic acid [27] (A, E)	C_9_H_16_O_4_	[M − H]^−^	30.60	188.10486	Organic acids
16	Cinnamic acidelluu [31] (B)	C_9_H_8_O_2_	[M + H]^+^	34.02	148.05243	Phenylpropanoids
17	Isomucronulatol [32] (A)	C_17_H_18_O_5_	[M + H]^+^	36.41	302.11542	Flavonoids
18	Calycosin [33] (A)	C_16_H_12_O_5_	[M + H]^+^	37.99	284.06847	Flavonoids
19	6-Methylcoumarin [25] (A, B)	C_10_H_8_O_2_	[M + H]^+^	38.64	160.05243	Phenylpropanoids
20	Formononetin [34] (A)	C_16_H_12_O_4_	[M − H]^−^	45.10	268.07356	Flavonoids
21	Truxinic acid [26] (B)	C_18_H_16_O_4_	[M − H]^−^	45.83	296.10486	Organic acids
22	6-Shogaol [31] (D)	C_17_H_24_O_3_	[M + H]^+^	46.55	276.17254	Gingerols
23	6-Gingerol [26] (D)	C_17_H_26_O_4_	[M − H]^−^	47.18	294.18311	Gingerols
24	Dibutyl phthalate [31]	C_16_H_22_O_4_	[M + H]^+^	53.91	278.15181	Organic acid esters
25	(9Z)-9-oleamide [31]	C_18_H_35_NO	[M + H]^+^	58.59	281.27187	Others

Notes: A: *Astragali radix*; B: *Cinnamomi ramulus*; C: *Paeoniae radix* Alba; D: *Zingiberis rhizoma* Recens; and E: *Jujubae fructus*.

**Table 2:** Typical biomarkers in plasma samples.

No.	*t*_R_ (min)	Observed *m*/*z*	Putative Identification	Formula	Trend
1	11.75	217.06559	Phenylacetylglycine	C_10_H_11_NO_3_	↓
2	3.64	172.06005	N-Acetyl-L-glutamate	C_7_H_11_NO_5_	↓
3	5.06	120.0806	N-Formiminoglycine	C_3_H_6_N_2_O_2_	↓
4	3.90	139.0498	Urocanate	C_6_H_6_N_2_O_2_	↓
5	3.45	193.03402	Citrate	C_6_H_8_O_7_	↓
6	6.20	86.06021	4-Aminobutanoate	C_4_H_9_NO_2_	↓
7	11.13	182.07001	Hippurate	C_9_H_9_NO_3_	↓
8	2.39	259.11371	sn-Glycero-3-phosphocholine	C_8_H_21_NO_6_P	↓
9	4.20	229.03172	(2S,3R)-3-Hydroxybutane-1,2,3-tricarboxylate	C_7_H_10_O_7_	↓
10	13.64	131.04818	Benzyl alcohol	C_7_H_8_O	↓
11	28.41	325.21107	Linoleate	C_18_H_32_O_2_	↑
12	5.37	229.03173	2-Methylcitrate	C_7_H_10_O_7_	↓
13	2.39	391.11028	D-Gluconic acid	C_6_H_12_O_7_	↓

Notes: Arrows indicate up regulation (↑) or down regulation (↓).

**Table 3:** Pharmacokinetic parameters of four compounds in plasma from CIA rats.

Constituent	*AUC*_(0–t)_ (mg/L·h)	*AUC*_(0–∞)_ (mg/L·h)	*C*_max_ (mg/L)	*T*_1/2_	*T*_max_	*MRT*_(0–t)_	*CL*	*Vd*
				(h)	(h)	(h)	(L/h/kg)	(L/kg)
calycosin	352.40 ± 17.01	559.24 ± 207.50	201.63 ± 89.40	6.99 ± 6.34	0.31 ± 0.12	2.63 ± 0.32	38,858.70 ± 10,417.32	317,500.32 ± 147,314.23
paeoniflorin	10,137.39 ± 1825.85	12,023.27 ± 2812.92	5143.50 ± 2302.67	4.83 ± 2.43	0.28 ± 0.13	3.28 ± 0.50	1751.11 ± 461.00	11,437.25 ± 4275.59
6-gingerol	142.11 ± 35.72	213.19 ± 116.05	131.54 ± 183.82	11.43 ± 16.99	0.23 ± 0.14	3.98 ± 1.01	111,579.16 ± 42,656.24	1,171,061.58 ± 1,081,344.82
formononetin	267.79 ± 53.79	337.08 ± 84.27	95.60 ± 36.14	2.76 ± 0.62	0.89 ± 1.53	3.02 ± 0.59	62,169.29 ± 13,883.83	244,433.41 ± 62,914.08

## Data Availability

This data can be found here: [https://data.mendeley.com/datasets/b3hfnytzm6/1] (accessed on 21 January 2026). The original contributions presented in this study are included in the article/Supplementary Materials. Further inquiries can be directed to the corresponding author.

## Supplementary Materials

The following supporting information can be downloaded at: https://www.mdpi.com/article/10.3390/ph19020236/s1, Figure S1. HPLC chromatograms of HGWD mixed reference substances (A), sample (B), sample without *Astragalus membranaceus* (C),sample without *Paeoniae Radix* Alba (D), sample without *Zingiberis Rhizoma* Recens (E). 1. Paeoniflorin; 2. Calycosin; 3. Formononetin; 4. 6-Gingerol. Table S1. Results of precision test of the four analytes in HGWD. Table S2. Results of stability test of the four analytes in HGWD. Table S3. Results of repeatability test of the four analytes in HGWD. Table S4. Results of accuracy test of the four analytes in HGWD. Table S5. Results of content of the four analytes in HGWD. Figure S2. HPLC chromatograms of mixed reference substances(A), sample(B). 1.Paeoniflorin; 2. Calycosin; 3. Formononetin; 4. 6-Gingerol. Paeoniflorin belongs to *Paeoniae Radix* Alba, Calycosin and Formononetin belongs to *Astragalus membranaceus*, 6-Gingerol belongs to *Zingiberis Rhizoma*. Table S6. List of selected multiple reaction monitoring parameters, declustering potential(DP), entrance potential (EP), collision energy (CE) and cell exit potential (CXP) for each analyte and IS. Figure S3. Typical chromatograms of blank plasma (A), blank plasma sample spiked with paeoniflorin, calycosin, formononetin and 6-Gingerol at LLOQ and IS (B) and plasma sample collected at 1.5 h after oral administration of HGWWD (C). (1) paeoniflorin, (2) calycosin, (3) formononetin, (4) 6-Gingerol, (5) geniposide (IS), (6) 4’-O-beta-Glucopyranosyl-5-O-Methylvisamminol (IS). Table S7. Summary of accuracy, precision, extraction recovery and matrix effect of the four analytes in rat plasma (*n* = 6). Table S8. Stability of the four analytes in rat plasma under different storage conditions (*n* = 3). Table S9. The quantitative analysis of calycosin, paeoniflorin, 6-gingerol, and formononetin in the plasma of rat. Table S10. List of chromatographic condition. Table S11. List of MS condition. Figure S4. The total ion chromatograms of HGWD intragastric administration plasma in positive and negative ion mode. (A) Positive ion mode; (B) Negative ion mode. Table S12. Identification of the chemical constituents of HGWD. Figure S5. TIC of positive and negative ion patterns of plasma samples. A: positive mode; B: negative mode. Table S13. Precision, repeatability and stability in the method validation of the metabolomic study in positive mode. Table S14. Precision, repeatability and stability in the method validation of the metabolomic study in negative mode. Table S15. The target prediction scores for each compound in HERB. Table S16. The target prediction scores for each compound in SWISS. Table S17. The target prediction scores for each compound in SEA.
