# Supplementary material for: Multi-Targeted Therapeutic Mechanisms of Huangqi Guizhi Wuwu Decoction Against Rheumatoid Arthritis: An Integrated Approach Combining Serum Pharmacochemistry, Network Pharmacology, Metabolomics, and Experimental Validation
Source: Pharmaceuticals (Basel). 2026 Jan 29;19(2):236. doi: 10.3390/ph19020236 (PMC12943270; doi:10.3390/ph19020236)
Supplement: Supplementary file 1 [file pharmaceuticals-19-00236-s001.zip › pharmaceuticals-4084047-supplementary.pdf]

Supplementary Material

1.Preparation of Huangqi Guizhi Wuwu Decoction (HGWD)

HGWD was prepared based on the original formulation recorded in the Synopsis of Prescriptions of the Golden Chamber, and herbs were purchased from Hebei Tiancheng Pharmaceutical (Hebei, China). Five ingredients -- Astragali Radix (90g), Paeoniae Radix Alba (90g), Cinnamomi Ramulus (90g), Zingiberis Rhizoma Recens (180g), Jujubae Fructus (90g)--were soaked in 12 L of pure water for 1 hour, refluxed for an additional hour. The resulting extract was filtered through gauze to collect the filtrate. The filtrate was then mixed with another 12L of water, refluxed for 1 hour, and filtered again. The two filtrates were combined, concentrated to an extract under reduced pressure, and subsequently diluted and re-dissolved to achieve a final concentration of 1.5 g/mL for the HGWD extracts.

2.The quantitative analysis of calycosin, paeoniflorin, 6-gingerol, and formononetin in HGWD

2.1 Preparation method of the analytical sample

Take 1 mL of HGWD extracts(1.5 g/mL), place it in a 10 mL volumetric flask, and dilute to the mark with 80% methanol to obtain the analytical sample.

2.2 HPLC method for calycosin, paeoniflorin, 6-gingerol, and formononetin analysis in HGWD

Chromatographic separation was carried out using an Thermo U3000 HPLC system equipped with a Agilent ZORBAX SB-C18 column (4.6×250 mm, 5 μm). The mobile phase consisted of (A) 0.1% formic acid in water and (B) acetonitrile, with a gradient elution as follows: 0-55 min,10%-35% B; 55-65 min,35%-48% B; 65-66 min,48%-10% B; 66-75 min,10% B. The flow rate was set at 1.0 mL/min, and the column temperature was maintained at 30°C

2.3 The validation results for the analytical methods in HGWD

Method validation was performed in accordance with the guidelines outlined in the Chinese Pharmacopoeia (2020 edition), evaluating specificity, precision, stability, repeatability, and accuracy .

2.3.1 Specificity

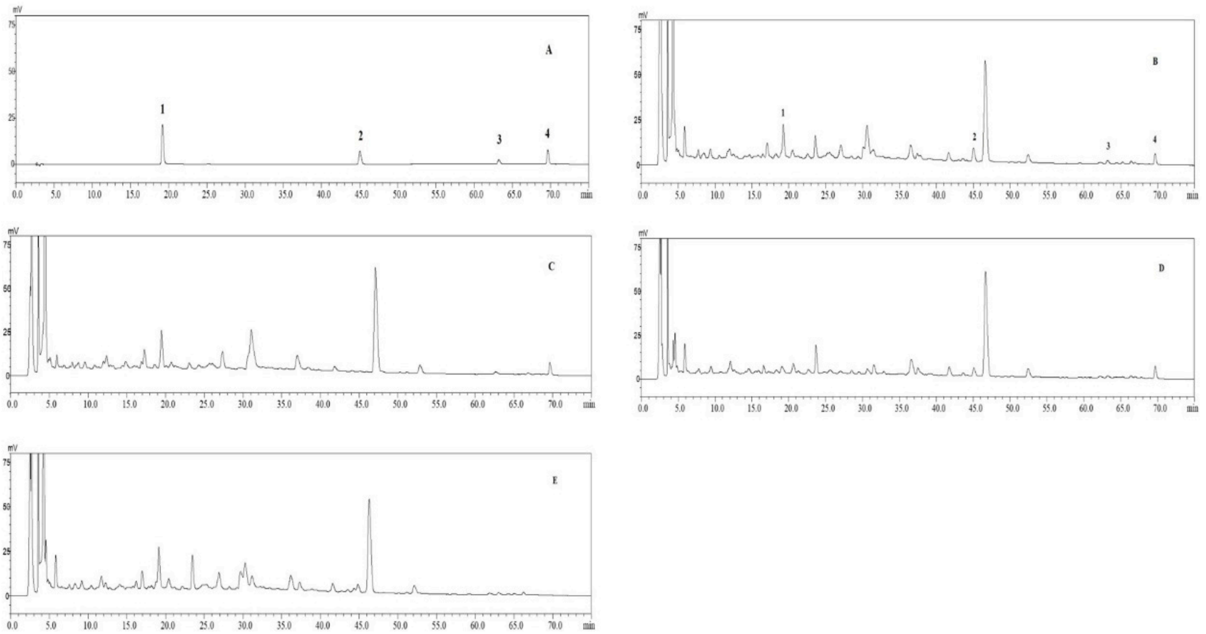

Figure S1. HPLC chromatograms of HGWD

mixed reference substances(A),sample(B),sample without Astragalus membranaceus (C),sample without Paeoniae Radix Alba (D) ,sample without Zingiberis Rhizoma Recens (E).1.Paeoniflorin; 2.Calycosin; 3.Formononetin; 4. 6-Gingerol

2.3.2 Precision

Table S1. Results of precision test of the four analytes in HGWD

Analytes	Area	RSD(%)
paeoniflorin	326.61	0.99
	327.47	
	325.66	
	330.54	
	332.92	
	333.06	
calycosin	305.18	0.53
	304.97	
	304.47	
	305.97	
	306.20	

	309.01	
formononetin	94.26<br>93.92<br>92.96<br>94.77<br>94.68<br>92.38	1.03
6-Gingerol	154.62<br>153.91<br>156.97<br>160.23<br>155.59<br>158.73	1.56

2.3.3 Stability

Table S2. Results of stability test of the four analytes in HGWD

Analytes	Time(h)	Area	RSD(%)
paeoniflorin	0	442.01	1.95
	2	448.65	
	4	443.53	
	6	431.86	
	8	432.07	
	12	426.49	
calycosin	0	250.70	2.48
	2	259.87	
	4	264.40	
	6	265.56	
	8	266.47	
	12	268.54	

formononetin	0	102.55	1.05
	2	103.65	
	4	102.49	
	6	105.09	
	8	102.86	
	12	104.51	
6-Gingerol	0	155.48	1.61
	2	157.03	
	4	156.90	
	6	160.56	
	8	159.59	
	12	162.15	

2.3.4 Repeatability

Table S3. Results of repeatability test of the four analytes in HGWD

Analytes	Content of test sample (μg/g)	average content (μg/g)	RSD(%)
paeoniflorin	3061.96	2988.11	2.60
	3079.35		
	2872.21		
	2993.62		
	2934.93		
	2986.63		
calycosin	57.65	57.53	1.69
	57.73		
	56.81		
	59.29		
	56.61		
	57.08		

formononetin	15.43<br>15.13<br>15.15<br>14.99<br>15.35<br>15.02	15.18	1.17
6-Gingerol	181.91<br>174.37<br>170.57<br>180.45<br>173.21<br>176.56	176.18	2.47

2.3.5 accuracy

Table S4. Results of accuracy test of the four analytes in HGWD

Analytes	$m_{original}$ (μg)	m_{added} (μg)	m_{found} (μg)	Recovery (%)	Average (%)	RSD (%)
paeoniflorin	2241.09	2295.00	4432.97	95.51	97.31	1.90
	2241.09	2295.00	4442.70	95.93		
	2241.09	2295.00	4446.32	96.09		
	2241.09	2295.00	4482.56	97.67		
	2241.09	2295.00	4498.21	98.35		
	2241.09	2295.00	4543.89	100.34		
calycosin	43.15	42.24	84.43	97.73	98.73	2.10
	43.15	42.24	84.99	99.06		
	43.15	42.24	84.83	98.68		
	43.15	42.24	85.68	100.70		
	43.15	42.24	85.78	100.92		
	43.15	42.24	83.40	95.29		
formononetin	7.59	7.71	15.25	99.35	100.60	2.69

	11.39	11.61	23.25	102.15		
	11.39	11.61	23.01	100.09		
	11.39	11.61	22.48	95.52		
	11.39	11.61	23.37	103.19		
	11.39	11.61	23.2	101.72		
6-Gingerol	153.30	137.10	290.54	100.11	101.96	1.31
	153. 30	137.10	292.70	101.68		
	153. 30	137.10	291.62	100.89		
	153. 30	137.10	294.42	102.94		
	153. 30	137.10	295.47	103.70		
	153. 30	137.10	293.72	102.42		

2.4 The results of quantitative analysis of calycosin, paeoniflorin, 6-gingerol, and formononetin in HGWD

Table S5. Results of content of the four analytes in HGWD

Analytes	Content of test sample (μg/g)	Average content (μg/g)
paeoniflorin	2950.35	2962.38
	3255.85	
	2680.93	
calycosin	56.06	56.43
	61.84	
	51.37	
formononetin	14.62	14.50
	15.05	
	13.84	
6-Gingerol	172.54	170.91
	188.69	
	151.50	

3. Assign peaks of calycosin, paeoniflorin, 6-gingerol, and formononetin on HPLC to the corresponding plants.

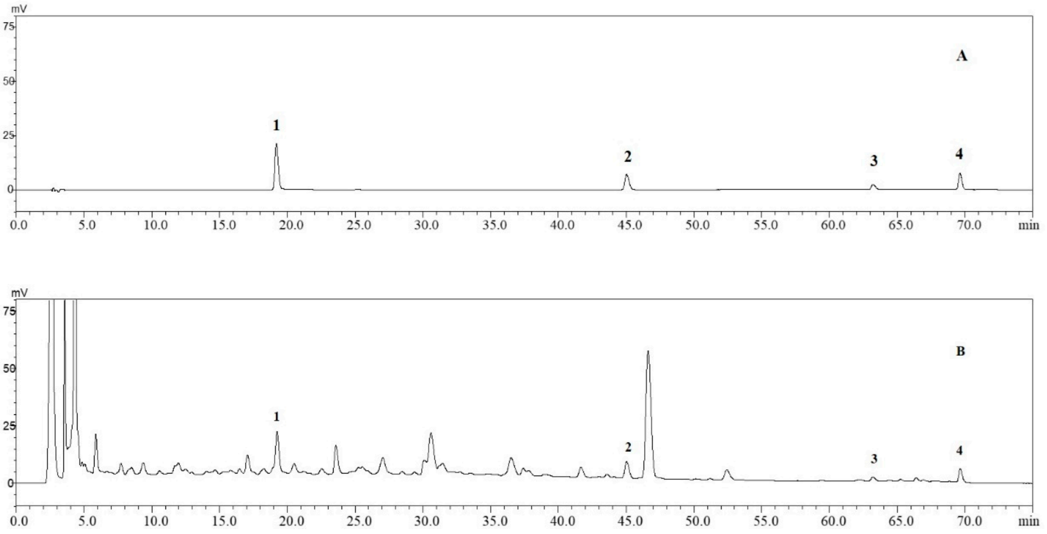

Figure S2. HPLC chromatograms of mixed reference substances(A),sample(B)

1.Paeoniflorin; 2.Calycosin; 3.Formononetin; 4. 6-Gingerol

Paeoniflorin belongs to Paeoniae Radix Alba, Calycosin and Formononetin belongs to Astragalus membranaceus, 6-Gingerol belongs to Zingiberis Rhizoma .

4.Pharmacokinetic Study

4.1 HPLC-MS/MS method for pharmacokinetics

Chromatographic separation was carried out using an Shimadzu 20A HPLC system equipped with a Waters C_{18} column (2.1×100 mm, 3.5 μm). The mobile phase consisted of (A) 0.1% formic acid in water and (B) acetonitrile, with a gradient elution as follows: 0-5 min, 10%-20% B; 5-6 min, 20%-50% B; 6-8 min, 50%-60% B; 8-10 min, 60%-88% B. The flow rate was set at 0.3 mL/min, the injection volume was 5 μL, and the column temperature was maintained at 35°C.

Mass spectrometric detection was performed on an Shimadzu API 3200 MD 3Q mass spectrometer with an electrospray ionization (ESI) source in both positive and negative ion segmeented alternating modes. The ion source temperature was maintained at 450 °C. The ion spray voltage was set at 4500 V in positive ion mode and -5500 V in negative ion mode. Nitrogen was used as the nebulizer gas, auxiliary heating gas, curtain gas, and collision gas, with flow rates of 45, 50, 20, and 5 psi, respectively. Multiple reaction monitoring (MRM) mode was applied for data acquisition. The optimized parameters and ion transitions for the five target analytes and two internal standards are summarized in Table S6.

Table S6. List of selected multiple reaction monitoring parameters, declustering potential(DP), entrance potential (EP), collision energy (CE) and cell exit potential (CXP) for each analyte and IS

Analyte	Ion Monitoring Mode	Q1 mass（Da）	Q3 mass（Da）	DP	EP	CE
geniposide	Negative（0~6.487min）	433.1	225.0	-42	-49.90	-22
paeoniflorin		525.1	121.0	-52	-52.48	-23
4'-*O*-beta-Glucopyranosyl-5-*O*-Methylvisamminol	Positive（6.487~7.39min）	453.4	291.4	79	24.73	32
calycosin	Negative（7.39~8.24min）	283.1	210.8	-71	-45.66	-48
formononetin		266.9	252.1	-58	-41.82	-16
6-Gingerol	Positive（8.24~10min）	277.3	177.3	60	18.23	16

4.2 The validation results for the analytical methods in the plasma of rat

Validation of the HPLC-MS/MS methodology was conducted in accordance with the United States Food and Drug Administration (FDA) Bioanalytical Method Validation Guidance for Industry, encompassing the evaluation of selectivity, linearity, precision, accuracy, matrix effects, recovery efficiency, and stability characteristics.

4.2.1 Specificity

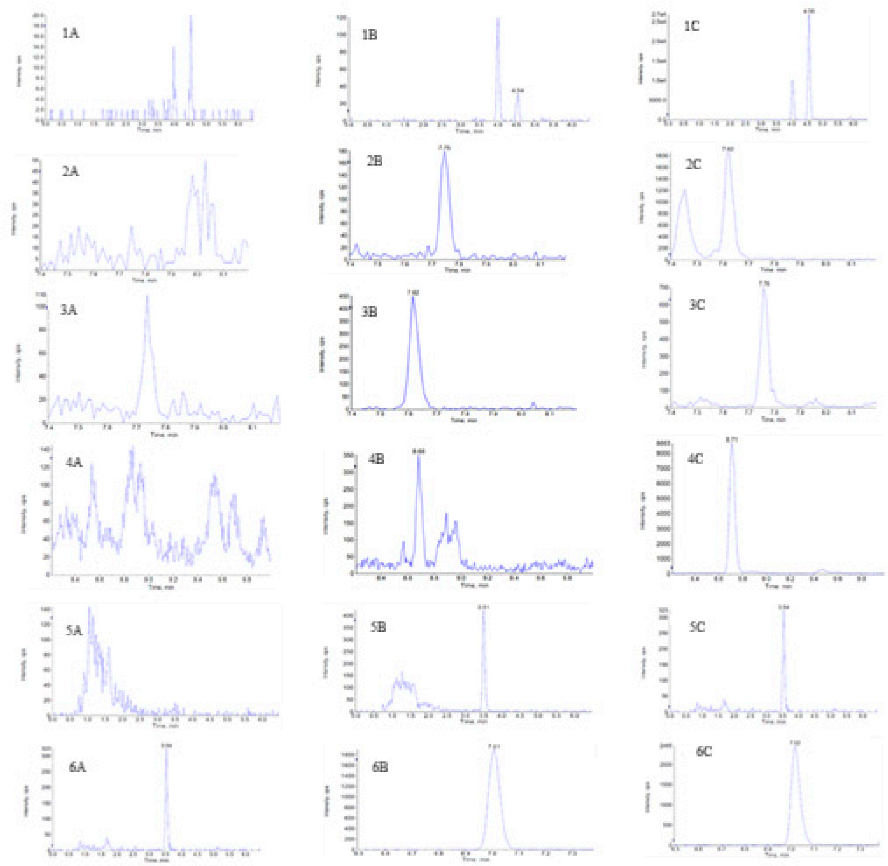

Figure S3.Typical chromatograms of blank plasma (A), blank plasma sample spiked with paeoniflorin, calycosin, formononetin and 6-Gingerol at LLOQ and IS(B) and plasma sample collected at 1.5 h after oral administration of HGWWD(C). (1) paeoniflorin, (2) calycosin, (3) formononetin , (4) 6-Gingerol, (5) geniposide (IS), (6) 4'-O-beta-Glucopyranosyl-5-O-Methylvisamminol (IS)

4.2.2 Precision , accuracy, extraction recovery and matrix effect

Table S7. Summary of accuracy, precision, extraction recovery and matrix effect of the four analytes in rat plasma (n = 6).

Analytes	Concentration (ng/mL)	Intra-day RSD(%)	Inter-day RSD(%)	Accuracy (RE %)	Recovery (%,mean±SD)	Matrix effect (%,mean±SD)
	20	5.7	8.9	9.1	86.7±5.2	96.9±10.0
paeoniflorin	80	10.8	11.7	5.4	93.9±5.5	92.2±8.4
	480	4.4	7.9	4.3	89.7±7.2	91.2±4.1
calycosin	40	13.5	9.8	-1.2	91.6±6.9	95.5±12.1

	160	4.4	9.7	-0.1	94.3±2.2	89.9±1.3
	960	2.9	9.3	2.8	90.1±7.6	87.4±7.5
	12.5	12.9	10.0	8.2	81.8±5.4	91.8±7.0
formononetin	50	7.4	7.7	2.4	89.9±6.4	94.0±5.1
	300	6.2	7.7	5.7	89.9±6.5	87.9±5.9
	2	7.0	9.6	-0.5	91.7±6.3	92.3±5.2
6-Gingerol	8	7.1	10.2	2.6	89.9±6.7	89.8±6.8
	48	3.8	5.0	2.4	87.6±10.0	92.8±3.7

4.2.3 Stability

Table S8. Stability of the four analytes in rat plasma under different storage conditions (n = 3).

Analytes	Concentration (ng/mL)	Room temperature for 4 h		Processed samples in autosampler vials for 12 h		Three freeze-thaw cycles		-80°C for 14 days	
		RE (%)	RSD (%)	RE (%)	RSD (%)	RE (%)	RSD (%)	RE (%)	RSD (%)
	20	0.1	1.9	4.3	7.1	10.2	13.0	6.4	5.4
paeoniflorin	80	14.9	6.2	14.0	4.0	13.8	1.3	3.9	1.8
	480	10.8	12.5	3.5	4.0	6.9	5.5	-2.7	10.3
	40	4.8	9.6	10.9	8.1	-1.0	9.5	7.2	1.8
calycosin	160	0.6	4.3	4.4	8.2	0.8	3.0	1.5	8.6
	960	-0.2	8.4	-5.6	7.7	-6.3	4.4	-1.6	6.2
	12.5	12.4	5.6	8.0	10.3	11.2	9.3	6.0	8.6
formononetin	50	14.1	8.2	2.1	6.3	8.6	5.7	12.1	6.4
	300	6.0	7.7	4.5	8.1	4.1	2.8	7.3	6.9
	2	-4.8	3.1	9.7	12.0	-5.6	4.0	10.3	10.1
6-Gingerol	8	4.4	11.0	12.1	10.3	-0.6	6.5	9.3	10.7
	48	9.7	1.7	9.3	3.1	2.5	3.5	-5.0	4.7

4.3 The quantitative analysis of calycosin, paeoniflorin, 6-gingerol, and formononetin in the plasma of rat

Table S9. The quantitative analysis of calycosin, paeoniflorin, 6-gingerol, and formononetin in the plasma of rat

(h)	paeoniflorin（ng/mL）	calycosin（ng/mL）	formononetin（ng/mL）	6-Gingerol（ng/mL）
0.083	3969.33	61.60	62.77	240.09
0.25	4603.50	155.35	72.45	129.35
0.5	3834.67	144.34	85.96	29.34
0.75	2848.83	117.24	71.96	20.17
1	2003.83	85.14	53.65	16.16
1.5	1763.67	67.69	44.42	14.83
2	1396.17	46.94	37.97	14.49
3	955.13	38.03	32.88	10.42
4	873.58	32.58	33.12	16.52
6	491.72	26.29	24.49	8.10
8	393.28	20.41	8.50	7.54
12	215.53	ND	ND	4.39

5.HPLC-Q-TOF-MS/MS method for serum pharmacochemistry analysis

Chromatographic separation was achieved on an Agilent Poroshell 120 SB-AQ column (100mm×4.6mm×2.7μm) at the oven temperature of 30°C. The mobile phase consisted of 0.1% formic acid in water (A) and 0.1% formic acid in acetonitrile (B) with a flow rate of 0.4 mL/min. The optimized gradient elution program was as followed table. The injection volume was 5 μL, and the temperature of the autosampler tray was constantly kept at 4°C.

Table S10. List of chromatographic condition

Time (min)	Phase A (%)	Phase B (%)
0~10	95~90	5~10
10~15	90~88	10~12
15~25	88~75	12~25

25~35	75~70	25~30
35~40	70~55	30~45
40~60	55~10	45~90
60~60.01	10~95	90~5
60.01~70	95~95	5~5

Mass spectrometric detection was accomplished by an electrospray ionization (ESI) source operated in both positive and negative modes with a scanning range of m/z 50-1500Da. The optimized parameters were as follows:

Table S11. List of MS condition

**ESI Parameters**	
Ionspray Voltage (V)	5500V(+)/4500(-)
Temperature (℃)	550
Ion Source Gas1 (psi)	50
Ion Source Gas2 (psi)	50
Curtain Gas (psi)	30

Parameters of TOF MS	
Declustering Potential (V)	±80V
Collision Energy (V)	±10V

Parameters of TOF MS/MS	
Declustering Potential (V)	±80V
Collision Energy (V)	±35V

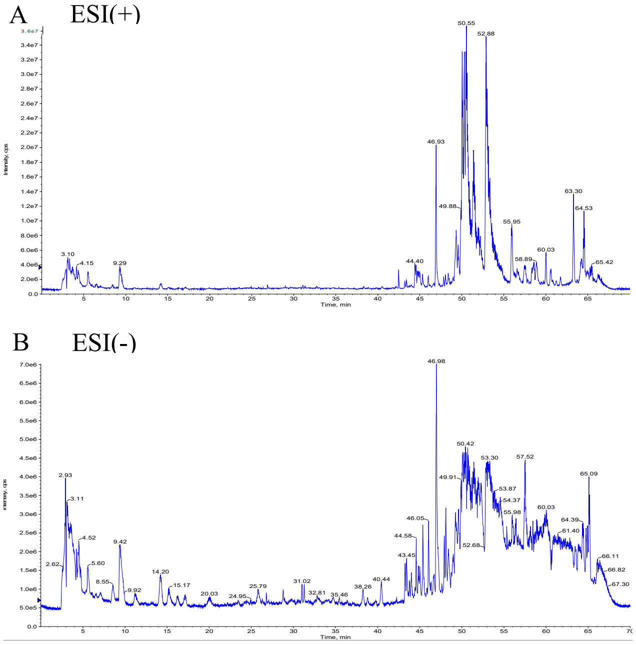

Figure S4.The total ion chromatograms of HGWD intragastric administration plasma in positive and negative ion mode. (A) Positive ion mode; (B) Negative ion mode

Table S12. Identification of the chemical constituents of HGWD.

**Peak No.**	Identification	Formula	Adduct	t_R (min)	Calculated (m/z)	Classification	Source
1	L(+)-Arginine	$C_6H_{14}N_4O_2$	$[M+H]^+$	2.89	174.11168	Amino acids	A, C, D, E
2	Choline	$C_5H_{13}NO$	$[M+H]^+$	2.91	103.09971	Alkaloids	C
3	Asparagine	$C_4H_8N_2O_3$	$[M+H]^+$	2.94	132.05349	Amino acids	C
4	L-Threonine	$C_4H_9NO_3$	$[M+H]^+$	2.95	119.05824	Amino acids	A, D, E
5	DL-Proline	$C_5H_9NO_2$	$[M+H]^+$	3.12	115.06333	Amino acids	A, D, E
6	Sucrose	$C_{12}H_{22}O_{11}$	$[M+H]^+$	3.18	342.11621	Saccharide	A, B, C, E
7	Betaine	$C_5H_{11}NO_2$	$[M+H]^+$	3.28	117.07898	Alkaloids	A
8	Desbenzoyl-paeoniflorin	$C_{16}H_{24}O_{10}$	$[M-H]^-$	4.1	376.13695	Monoterpene glycosides	C
9	Adenine	$C_5H_5N_5$	$[M+H]^+$	4.21	135.0545	Alkaloids	A, E
10	Nicotinic acid	$C_6H_5NO_2$	$[M+H]^+$	4.21	123.03203	Organic acids	A
11	Tyrosine	$C_9H_{11}NO_3$	$[M+H]^+$	4.4	181.07389	Amino acids	A, B, C, D, E
12	Nicotinamide	$C_6H_6N_2O$	$[M+H]^+$	4.47	122.04801	Alkaloids	E
13	Uridine	$C_9H_{12}N_2O_6$	$[M-H]^-$	4.49	244.06954	Nucleosides	B, C
14	Phenylalanine	$C_9H_{11}NO_2$	$[M+H]^+$	5.59	165.07898	Amino acids	A
15	Guanosine	$C_{10}H_{13}N_5O_5$	$[M-H]^-$	5.72	283.09167	Nucleosides	A
16	2-Methoxybenzoic acid	$C_8H_8O_3$	$[M+H]^+$	5.88	152.04734	Organic acids	B
17	Guanine	$C_5H_5N_5O$	$[M+H]^+$	5.94	151.04941	Alkaloids	E
18	Glucopyranos-L-paeonisuffrone	$C_{16}H_{24}O_9$	$[M-H]^-$	6.17	360.14203	Monoterpene glycosides	C

19	Adenosine	$C_{10}H_{13}N_5O_4$	$[M+H]^+$	6.22	267.09675	Nucleosides	A
20	1-O-galloyl-beta-D-glucose	$C_{13}H_{16}O_{10}$	$[M-H]^-$	6.94	332.07435	Phenols	C
21	Gallic acid	$C_7H_6O_5$	$[M-H]^-$	7.07	170.02152	Organic acids	B, C, E
22	1'-O-galloylsucrose	$C_{19}H_{26}O_{15}$	$[M-H]^-$	7.68	494.12717	Saccharide	C
23	Markhamioside F	$C_{18}H_{26}O_{12}$	$[M-H]^-$	9.04	434.14243	Phenols	A
24	Vanillic acid	$C_8H_8O_4$	$[M-H]^-$	9.46	168.04226	Organic acids	A, B, C
25	Tryptophan	$C_{11}H_{12}N_2O_2$	$[M+H]^+$	9.53	204.08988	Amino acids	A, D, E
26	Diethyl phthalate	$C_{12}H_{14}O_4$	$[M+H]^+$	9.66	222.08921	Organic acid esters	E
27	Lactinolide	$C_{10}H_{16}O_4$	$[M+H]^+$	9.85	200.10486	Monoterpenoids	C
28	Succinoadenosine	$C_{14}H_{17}N_5O_8$	$[M-H]^-$	11.22	383.10771	Nucleosides	A
29	Propyl gallate	$C_{10}H_{12}O_5$	$[M-H]^-$	12.18	212.06847	Monoterpene glycosides	C
30	8-O-galloyl desbenzoylpaeoniflorin	$C_{23}H_{28}O_{14}$	$[M-H]^-$	14.30	528.14791	Monoterpene glycosides	C
31	4-Hydroxybenzoic acid	$C_7H_6O_3$	$[M+H]^+$	14.59	138.03169	Organic acids	B, C
32	Methyl gallate	$C_8H_8O_5$	$[M-H]^-$	15.84	184.03717	Organic acid esters	C
33	Oxypaeoniflora	$C_{23}H_{28}O_{12}$	$[M+H]^+$	17.38	496.15808	Monoterpene glycosides	C
34	Dihydrocinnacasside	$C_{15}H_{20}O_8$	$[M-H]^-$	18.44	328.11582	Phenylpropanoids	B
35	Paeonol	$C_9H_{10}O_3$	$[M+H]^+$	18.51	166.06299	Phenols	B, C
36	Catechin	$C_{15}H_{14}O_6$	$[M-H]^-$	18.66	290.07904	Flavonoids	C
37	Protocatechuic acid	$C_7H_6O_4$	$[M-H]^-$	20.84	154.02661	Organic acids	A, C, D, E

38	β-gentiobiosyl paeoniflorin	$C_{29}H_{38}O_{16}$	$[M+H]^+$	21.44	642.21599	Monoterpene glycosides	C
39	Albiflorin	$C_{23}H_{28}O_{11}$	$[M+H]^+$	22.01	480.16316	Monoterpene glycosides	C
40	Albiflorin C	$C_{17}H_{18}O_{6}$	$[M+H]^+$	22.03	318.11034	Monoterpene glycosides	C
41	Isomaltopaeoniflorin	$C_{29}H_{38}O_{16}$	$[M-H]^-$	22.73	642.21599	Monoterpene glycosides	C
42	Ethyl gallate	$C_{9}H_{10}O_{5}$	$[M-H]^-$	22.99	198.05282	Organic acid esters	C
43	Paeoniflorin	$C_{23}H_{28}O_{11}$	$[M-H]^-$	23.49	480.16316	Monoterpene glycosides	C
44	Mudanpioside E	$C_{24}H_{30}O_{13}$	$[M-H]^-$	23.49	526.16864	Monoterpene glycosides	C
45	Paeonilactone B	$C_{10}H_{12}O_{4}$	$[M+H]^+$	23.58	196.07356	Monoterpene glycosides	C
46	Coniferaldehyde	$C_{10}H_{10}O_{3}$	$[M+H]^+$	23.59	178.06299	Essential oil	B
47	4-Hydroxycinnamic acid	$C_{9}H_{8}O_{3}$	$[M-H]^-$	26.65	164.04734	Phenylpropanoids	B
48	Syringaldehyde	$C_{9}H_{10}O_{4}$	$[M+H]^+$	27.18	182.05791	Essential oil	A
49	Cinnacasside E	$C_{25}H_{38}O_{11}$	$[M+H]^+$	27.93	514.24141	Phenylpropanoids	B
50	Calycosin-7-O-β-D-glucoronide	$C_{22}H_{22}O_{10}$	$[M-H]^-$	27.94	446.1213	Flavonoids	A
51	Ferulic Acid	$C_{10}H_{10}O_{4}$	$[M-H]^-$	28.31	194.05791	Phenylpropanoids	A, B
52	Truxinic acid	$C_{18}H_{16}O_{4}$	$[M-H]^-$	45.86	296.10486	Phenylpropanoids	B
53	Rutin	$C_{27}H_{30}O_{16}$	$[M+H]^+$	28.52	610.15339	Flavonoids	E
54	Naringenin	$C_{15}H_{12}O_{5}$	$[M+H]^+$	28.60	272.06847	Flavonoids	C
55	Galloylpaeoniflorin	$C_{30}H_{32}O_{15}$	$[M-H]^-$	28.86	632.17412	Monoterpene glycosides	C

56	Kaempferol-3-O-rutinoside	$C_{27}H_{30}O_{15}$	$[M+H]^+$	29.66	594.15847	Flavonoids	A, C, E
57	Ellagic acid	$C_{14}H_6O_8$	$[M-H]^-$	29.76	302.00627	Phenols	C
58	Cosmosiin	$C_{21}H_{20}O_{10}$	$[M+H]^+$	29.86	432.10565	Flavonoids	A
59	Lactiflorin	$C_{23}H_{26}O_{10}$	$[M-H]^-$	30.03	462.1526	Monoterpene glycosides	C
60	Coumarin	$C_9H_6O_2$	$[M+H]^+$	30.19	146.03678	Phenylpropanoids	A, B
61	Azelaic acid	$C_9H_{16}O_4$	$[M-H]^-$	30.59	188.10486	Organic acids	A, E
62	Mudanpioside D	$C_{24}H_{30}O_{12}$	$[M-H]^-$	30.97	510.17373	Monoterpene glycosides	C
63	Kaempferol	$C_{16}H_{12}O_6$	$[M-H]^-$	31.22	300.06339	Flavonoids	B
64	Poncirin	$C_{28}H_{34}O_{14}$	$[M-H]^-$	33.41	594.19486	Flavonoids	A
65	Mudanpioside H	$C_{30}H_{32}O_{14}$	$[M-H]^-$	33.83	616.17921	Monoterpene glycosides	C
66	(Z) -(1S,5R) -β-pinene-10-yl-β-nethoside	$C_{21}H_{34}O_{10}$	$[M-H]^-$	33.90	446.2152	Glucoside	C
67	Calycosin-7-O-β-D- (6″-acetyl)-alkyl polyglucoside	$C_{24}H_{24}O_{11}$	$[M+H]^+$	33.92	488.13186	Flavonoids	A
68	Trans-Cinnamic acid	$C_9H_8O_2$	$[M+H]^+$	34.14	148.05243	Phenylpropanoids	B
69	Muanpioside C	$C_{30}H_{32}O_{13}$	$[M-H]^-$	34.45	600.18429	Glucoside	C
70	(6aR, 11aR)-3-hydroxy-9,l0-diMethoxy pterocarpan-7-O-β-D-glucoside	$C_{23}H_{26}O_{10}$	$[M+H]^+$	34.71	462.1526	Flavonoids	A
71	Isomucronulatol	$C_{17}H_{18}O_5$	$[M+H]^+$	35.76	302.11542	Flavonoids	A
72	7,2'-dihydroxy-3',4'-dimethoxyisoflavane-7-O-glucoside	$C_{23}H_{28}O_{10}$	$[M-H]^-$	35.77	464.16825	Flavonoids	A
73	Calycosin	$C_{16}H_{12}O_5$	$[M-H]^-$	37.99	284.06847	Flavonoids	A

74	6-Methylcoumarin	$C_{10}H_8O_2$	$[M+H]^+$	38.68	160.05243	Phenylpropanoids	A, B
75	Mudanpioside B	$C_{31}H_{34}O_{14}$	$[M-H]^-$	40.74	630.19486	Glucoside	C
76	6′-O-acetyl ononin	$C_{24}H_{24}O_{10}$	$[M+H]^+$	41.41	472.13695	Flavonoids	A
77	Astragaloside VI	$C_{47}H_{78}O_{19}$	$[M+H]^+$	41.49	946.51373	Triterpenoid saponins	A
78	Benzoylpaeoniflorin	$C_{30}H_{32}O_{12}$	$[M+H]^+$	41.69	584.18938	Monoterpene glycosides	C
79	Butylated Hydroxytoluene	$C_{15}H_{24}O$	$[M+H]^+$	43.17	220.18272	Monoterpenoids	C
80	Agroastragaloside IV	$C_{49}H_{80}O_{20}$	$[M+H]^+$	43.40	988.5243	Triterpenoid saponins	A
81	Betulonicacid	$C_{30}H_{46}O_3$	$[M+H]^+$	44.00	454.3447	Triterpenoids	B
82	23-Hydroxybetulinic acid	$C_{30}H_{48}O_4$	$[M+H]^+$	44.00	472.35526	Triterpenoids	C
83	Astragaloside IV	$C_{41}H_{68}O_{14}$	$[M+H]^+$	44.00	784.46091	Triterpenoid saponins	A
84	Formononetin	$C_{16}H_{12}O_4$	$[M-H]^-$	45.13	268.07356	Flavonoids	A
85	Oleanolic aldehyde	$C_{30}H_{48}O_2$	$[M+H]^+$	45.42	440.36543	Triterpenoids	C
86	SoyasaponinI	$C_{48}H_{78}O_{18}$	$[M+H]^+$	45.44	942.51882	Triterpenoid saponins	A
87	Astragaloside B	$C_{35}H_{58}O_9$	$[M+H]^+$	45.49	622.40808	Triterpenoid saponins	A
88	Astragaloside II	$C_{43}H_{70}O_15$	$[M+H]^+$	45.51	826.47147	Triterpenoid saponins	A
89	Methylnissolin	$C_{17}H_{16}O_5$	$[M+H]^+$	45.58	300.09977	Flavonoids	A
90	Agroastragaloside III	$C_{51}H_{82}O_{21}$	$[M+H]^+$	45.71	1030.53486	Triterpenoid saponins	A
91	6-Gingerol	$C_{17}H_{26}O_4$	$[M-H]^-$	46.57	294.18311	Gingerols	D
92	O-Anisaldehyde	$C_8H_8O_2$	$[M+H]^+$	46.58	136.05243	Essential oil	B

93	Ethyl cinnamate	$C_{11}H_{12}O_2$	$[M+H]^+$	46.58	176.08373	Organic acid esters	B
94	6-Shogaol	$C_{17}H_{24}O_3$	$[M+H]^+$	46.58	276.17254	Gingerols	D
95	Astragaloside I	$C_{45}H_{72}O_{16}$	$[M+H]^+$	48.93	868.48204	Triterpenoid saponins	A
96	8-Shogaol	$C_{19}H_{28}O_3$	$[M+H]^+$	50.32	304.20385	Gingerols	D
97	Palbinone	$C_{22}H_{30}O_4$	$[M+H]^+$	50.68	358.21441	Triterpenoids	C
98	Dibutyl phthalate	$C_{16}H_{22}O_4$	$[M+H]^+$	53.94	278.15181	Organic acid esters	B
99	(9Z)-9-oleamide	$C_{18}H_{35}NO$	$[M+H]^+$	58.61	281.27187	Others	B

6 The detailed method description for metabolomics analysis

6.1 Preparation of samples

One hundred microliters of plasma were diluted with a threefold volume of methanol. The mixture was vortexed for 5 min, centrifuged at 4 °C and 12,000 rpm for 15 min to obtain the supernatant, and then dried at 30 °C with N2. Finally, the samples were reconstituted with 100 µl of methanol, vortexed for 5 min and centrifuged (12,000 rpm at 4 °C for 10 min) to obtain the supernatant for injection. A quality control (QC) sample was prepared from 10 µL of each test sample.

6.2 The LC and MS conditions

The experiments were performed on an Agilent 1260 infinity liquid chromatography system (Agilent, Santa Clara, CA, USA) in tandem with a triple TOF 5600^+ quadrupole time-of-flight tandem mass spectrometer (Sciex, Redwood City, CA, USA) via an electrospray ionization (ESI) interface. The sample was separated on an Agilent poroshell 120 SB-AQ column (100 mm × 4.6 mm, 2.7 µm) at 30 °C. The mobile phase consists of water-formic acid (100:0.1, *v/v*, A) and acetonitrile-formic acid (100:0.1, *v/v*, B). The gradient program was optimized as follows: 0-5 min, 2-10% B; 5-10 min, 10-40% B; 10-20 min, 40-60% B; 20-30min, 60-90% B; 30-36 min, 90-2% B; 36-41 min, 2-2% B. The flow rate was 0.4 mL/min, and the injection volume was 4 µL. A flow rate of 0.5 mL/min and an injection volume of 4 µL were set.

The optimized parameters for the MS conditions were as follows: ion spray voltage, 5500V (+)/4500V (-); declustering potential, 80 V; the turbo spray temperature, 550 ºC; nebulizer gas (nitrogen), 50 psi; heater gas (nitrogen), 50 psi; and curtain gas (nitrogen), 30 psi. In the TOF-MS mode, the collision energy was 10 V. In the TOF-MS/MS mode, a typical information dependent acquisition (IDA) model was used with collision energy setting at 45 ± 15 V. The scan ranges in TOF-MS and TOF-MS/MS modes were both m/z 50-1500.

6.3 Analytical method validation for cell metabolomics

The extracted ion chromatographic peaks of six ions (*m/z*_tR: 147.1_2.1, 349.1_4.3, 201.1_7.2, 836.4_12.0, 457.3_23.0, and 408.3_32.0) in the positive ion mode and six ions (m/z_tR: 344.9_2.0,581.3_11.7, 527.3_19.2, 558.3_25.5, 395.2_29.2, and 116.9_34.8) in the negative ion mode were selected for method validation. The precision of the instrument was verified by 6 consecutive injections of the same quality control (QC) sample. To investigate the method repeatability, 6 independently processed parallel samples were prepared and analyzed. The stability of the samples was examined by injection of the same QC sample separately at 0, 4, 8,12,16 and 24h. The precision, repeatability, and sample stability of the developed method were all within acceptable limits (Tab. S2 and S3).

6.4 Data processing flow

The raw data were preprocessed using XCMS platform, and the generating data matrices were subjected to SIMCA software (version 14.1, Umetrics, Sweden) for multivariate statistical analysis. Metabolites were identified by comparing the m/z values and MS/MS spectra of detected compounds with those of commercially available standards and online metabolite databases (MetDNA). Potential biomarkers were screened based on stringent criteria: variable importance in projection (VIP) > 1.0 from the OPLS-DA model, fold change (FC) > 1.25 or < 0.8, and $p < 0.05$ (Student's t-test). Pathway enrichment analysis was carried out using MetaboAnalyst platform.

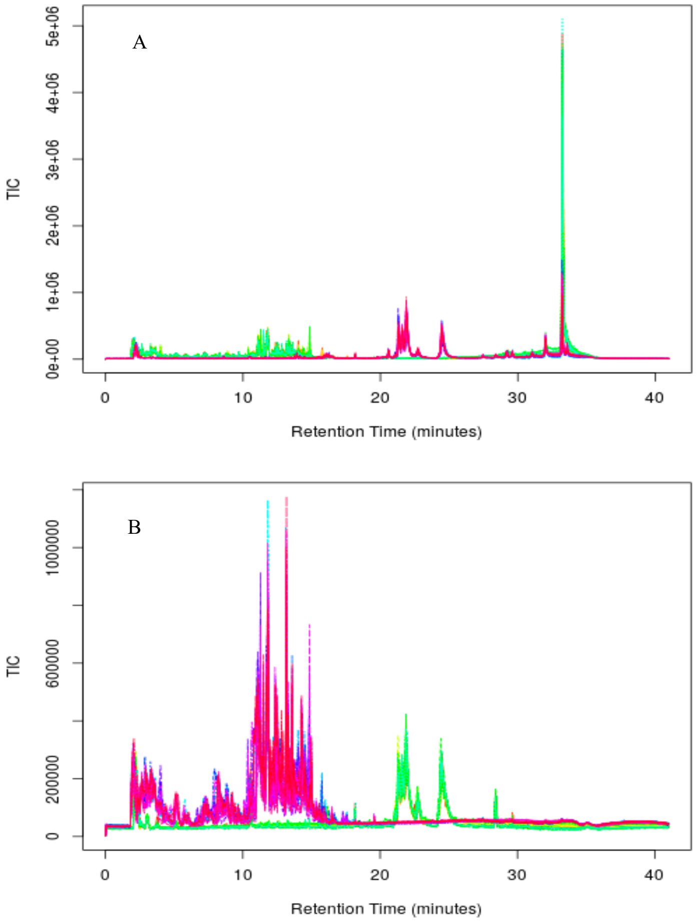

Figure S5. TIC of positive and negative ion patterns of plasma samples.A:positive mode;B:negative mode

Table S13. Precision, repeatability and stability in the method validation of the metabolomic study in positive mode.

*m/z*_t_R（min）	Precision (RSD%)		Repeatability (RSD%)		Stability (RSD%)	
	t_R	Intensity	t_R	Intensity	t_R	Intensity
147.1_2.1	0.0	6.3	0.1	4.6	0.1	3.5
349.1_4.3	0.1	5.0	0.1	2.0	0.1	5.5
201.1_7.2	0.1	2.4	0.1	2.2	0.1	5.0
836.4_12.0	0.1	4.6	0.1	5.8	0.0	5.3
457.3_23.0	0.1	5.7	0.1	4.7	0.1	3.9
408.3_32.0	0.1	8.3	0.1	5.6	0.0	8.2

Table S14. Precision, repeatability and stability in the method validation of the metabolomic study in negative mode.

*m/z*_t_R（min）	Precision (RSD%)		Repeatability (RSD%)		Stability (RSD%)	
	t_R	Intensity	t_R	Intensity	t_R	Intensity
344.9_2.0	0.6	4.6	0.7	3.6	0.7	4.9
581.3_11.7	0.2	2.2	0.1	3.6	0.3	4.1
527.3_19.2	0.0	9.3	0.1	2.7	0.1	5.2
558.3_25.5	0.1	6.5	0.4	6.8	0.1	4.0
395.2_29.2	0.2	8.8	0.2	6.5	0.2	6.6
116.9_34.8	0.0	5.5	0.1	8.0	0.1	4.2

7.The target prediction scores for each compound in network pharmacology

Table S15. The target prediction scores for each compound in HERB

6-Shogaol		Betaine		Choline		Protocatechuic acid		Tyrosine	
Target name	PubMed id	Target name	PubMed id	Target name	PubMed id	Target name	PubMed id	Target name	PubMed id
EIF2A	24215632	GLO1	31255657	SIGMAR1	31560162	PARK7	30326282	ANPEP	Database Mining
NFKB1	23612072	CHDH	31255657	CHRNA7	31560162	MTOR	30326282	AR	Database Mining
PPARG	23612072	FGF21	31120771	ACHE	Database Mining	NFE2L2	30326282	AVP	Database Mining
ALOX5	Database Mining	PPARA	31120771	ADH1A	Database Mining	BAX	30326282	B2M	Database Mining
FAS	Database Mining	SLC6A12	22053950	ADH1B	Database Mining	BCL2	30326282	BLMH	Database Mining
FASLG	Database Mining	LIPE	24819676	ADH4	Database Mining	CASP3	30326282	CDK5	Database Mining
BAX	Database Mining	PPP2CA	24819676	ADH5	Database Mining	CASP8	30326282	CPA1	Database Mining
BCL2	Database Mining	MECP2	26201319	ADH6	Database Mining	CASP9	30326282	CPA2	Database Mining
BCL2L1	Database Mining	PPARG	26201319	ADH7	Database Mining	IL1B	26603620	CPA3	Database Mining
CASP3	Database Mining	ABAT	Database Mining	BCHE	Database Mining	IL10	26603620	CPB1	Database Mining
CASP9	Database Mining	AKR1B1	Database Mining	BDNF	Database Mining	AKT1	20840540	CPB2	Database Mining
DDIT3	Database Mining	AQP1	Database Mining	BHMT	Database Mining	RHOB	20840540	CTSS	Database Mining
FASN	Database Mining	BCHE	Database Mining	CHAT	Database Mining	MMP2	20840540	DDC	Database Mining
FTL	Database Mining	CDC25B	Database Mining	CHKA	Database Mining	NFKB1	20840540	ENPEP	Database Mining

GCLC	Database Mining	CTSD	Database Mining	CHKB	Database Mining	RAS	20840540	FARSA	Database Mining
GCLM	Database Mining	GATM	Database Mining	CHRNA7	Database Mining	PRKCA	Database Mining	FUCA1	Database Mining
HMOX1	Database Mining	GH1	Database Mining	GCG	Database Mining	PRKCB	Database Mining	FUCA2	Database Mining
NFE2L2	Database Mining	GOT1	Database Mining	GRIN2A	Database Mining	PRKCG	Database Mining	GOT1	Database Mining
PPARG	Database Mining	GRIA1	Database Mining	LYZ	Database Mining	PRKCZ	Database Mining	GOT2	Database Mining
TRPV1	Database Mining	IGFBP1	Database Mining	PCYT1A	Database Mining	MGAM	Database Mining	HLA-A	Database Mining
CFLAR	Database Mining	LIPE	Database Mining	PLD1	Database Mining	CDK2	Database Mining	HLA-B	Database Mining
GDF15	Database Mining	MAOB	Database Mining	PLD2	Database Mining	MAOB	Database Mining	HLA-C	Database Mining
KEAP1	Database Mining	PTGS1	Database Mining	REN	Database Mining	ACHE	Database Mining	HLA-G	Database Mining
TBK1	Database Mining	PTGS2	Database Mining	S100A4	Database Mining	LYZ	Database Mining	LNPEP	Database Mining
AKR1B10	Database Mining	TNF	Database Mining	SLC5A5	Database Mining	PRSS3	Database Mining	LTA4H	Database Mining
GGTLC1	Database Mining	INMT	Database Mining	SLC22A1	Database Mining	DPEP1	Database Mining	OXT	Database Mining
		PPARG	Database Mining	SLC22A2	Database Mining	PRSS1	Database Mining	PAH	Database Mining
		GOT2	Database Mining	SUV39H1	Database Mining	PTGS1	Database Mining	CDK16	Database Mining
		GABRA1	Database Mining	TNF	Database Mining	CA2	Database Mining	CDK17	Database Mining
		GSK3B	Database Mining	SLC5A6	Database Mining			CDK18	Database Mining
		PRSS1	Database Mining	PCYT1B	Database Mining			CDK14	Database Mining
		ESR2	Database Mining	PTDSS1	Database Mining			PSMA1	Database Mining
		MAPK14	Database Mining	CLEC2B	Database Mining			PSMA3	Database Mining

CHEK1	Database Mining	PPARGC1A	Database Mining	PSMA4	Database Mining
AR	Database Mining	SLC44A1	Database Mining	PSMA5	Database Mining
ESR1	Database Mining	PLD3	Database Mining	PSMA6	Database Mining
CDK2	Database Mining	ETNK2	Database Mining	PSMA7	Database Mining
CCNA2	Database Mining	CHDH	Database Mining	PSMB1	Database Mining
TPI1	Database Mining	ETNK1	Database Mining	PSMB2	Database Mining
ACHE	Database Mining	GPCPD1	Database Mining	PSMB4	Database Mining
ME2	Database Mining	SLC44A2	Database Mining	PSMB5	Database Mining
NOS2	Database Mining	SLC5A7	Database Mining	PSMB6	Database Mining
DPP4	Database Mining	PLD4	Database Mining	PSMB7	Database Mining
PIM1	Database Mining	SLC44A3	Database Mining	PSMB10	Database Mining
		SLC5A12	Database Mining	PSMC1	Database Mining
		SLC5A8	Database Mining	PSMC2	Database Mining
		PHOSPHO1	Database Mining	PSMC3	Database Mining
		PLD6	Database Mining	PSMC4	Database Mining
		SLC44A5	Database Mining	PSMC5	Database Mining
		CHKB-CPT1B	Database Mining	PSMC6	Database Mining
				PSMD1	Database Mining
				PSMD2	Database Mining

PSMD3	Database Mining
PSMD4	Database Mining
PSMD5	Database Mining
PSMD7	Database Mining
PSMD8	Database Mining
PSMD9	Database Mining
PSMD10	Database Mining
PSMD11	Database Mining
PSMD12	Database Mining
PSMD13	Database Mining
PSME1	Database Mining
PSME2	Database Mining
PTEN	Database Mining
PVR	Database Mining
RNPEP	Database Mining
RPS27A	Database Mining
SLC3A2	Database Mining
TAT	Database Mining
TH	Database Mining

TPO	Database Mining
TPP2	Database Mining
TUBA4A	Database Mining
TUBA3C	Database Mining
TUBB2A	Database Mining
TUBG1	Database Mining
TYR	Database Mining
UBA52	Database Mining
UBB	Database Mining
UBC	Database Mining
TUBA1A	Database Mining
SLC7A5	Database Mining
YARS1	Database Mining
PSMF1	Database Mining
NPEPPS	Database Mining
PSMD6	Database Mining
FARSB	Database Mining
PSME3	Database Mining
PSMD14	Database Mining

TUBA1B	Database Mining
TUBB3	Database Mining
TUBB4A	Database Mining
TUBB4B	Database Mining
FARS2	Database Mining
TTLL5	Database Mining
PSME4	Database Mining
AGTPBP1	Database Mining
SLC7A8	Database Mining
TTLL1	Database Mining
TTLL3	Database Mining
TUBG2	Database Mining
TRHDE	Database Mining
YARS2	Database Mining
TUBD1	Database Mining
TUBE1	Database Mining
CPA4	Database Mining
ERAP1	Database Mining
TUBA8	Database Mining

ENOSF1	Database Mining
CPA6	Database Mining
RNPEPL1	Database Mining
LRRC47	Database Mining
AGBL5	Database Mining
ERAP2	Database Mining
CDK15	Database Mining
GGCT	Database Mining
TUBAL3	Database Mining
TUBB1	Database Mining
STMN4	Database Mining
TTLL2	Database Mining
TUBB6	Database Mining
TUBA1C	Database Mining
AGBL4	Database Mining
AOPEP	Database Mining
TUBA3E	Database Mining
SLC16A10	Database Mining
PSMB11	Database Mining

AGBL1	Database Mining
CPO	Database Mining
PSMA8	Database Mining
TTL	Database Mining
TTLL9	Database Mining
TTLL8	Database Mining
TUBB	Database Mining
LVRN	Database Mining
TTLL10	Database Mining
IL4I1	Database Mining
SLC6A19	Database Mining
AGBL3	Database Mining
TUBB2B	Database Mining
IYD	Database Mining
ARPC4-TTLL3	Database Mining

Table S16. The target prediction scores for each compound in SWISS

6-Shogaol		Choline		Nicotinamide		Phenylalanine		Protocatechuic acid	
Target name	Probability	Target name	Probability	Target name	Probability	Target name	Probability	Target name	Probability
ALOX5	1	Target	Probability	SIRT3	0.999487275	CACNA2D1	1	CA2	0.926267731
HTR1A	0.57566502	SLC5A7	1	SIRT2	0.999487275			CA7	0.926267731
								CA1	0.926267731
								CA6	0.926267731
								CA12	0.926267731
								CA14	0.926267731
								CA9	0.926267731
								CA4	0.926267731

Table S17. The target prediction scores for each compound in SEA

6-Methylcoumarin		6-Shogaol		Tyrosine		Truxinic acid		Protocatechuic acid		Phenylalanine		Nicotinamide		Luotonin		Dibutyl phthalate		Azelaic acid		Argininic acid	
Name	Max Tc	Name	Max Tc	Name	Max Tc	Name	Max Tc	Name	Max Tc	Name	Max Tc	Name	Max Tc	Name	Max Tc	Name	Max Tc	Name	Max Tc	Name	Max Tc
MAOB	0.5294	CNR1	0.5849	TH	0.5882	HDAC5	0.6154	CA14	1	RNPEP	0.5405	CYP11B1	0.5	TYR	0.5167	CYP1A2	0.5789	ENPEP	0.5217	CPN1	0.5938
CA14	0.5455	CNR2	0.5849	YARS1	0.6471	HDAC4	0.6154	CA12	1	LAP3	0.5333	CYP11B2	0.5			CNR2	0.5	CYP4F2	0.6		
CA12	0.6	ALOX5	1	PTPRA	0.5455	HDAC7	0.6154	CA1	1	ANPEP	0.5405	CYP3A4	0.5			CYP2C19	0.5758	FABP3	0.5217		
CA3	0.5172	TRPV1	1	PPARG	0.6	HDAC9	0.6154	CA3	0.5652	CTSC	0.5278	HDAC1	0.5			STS	0.5128	FOLH1	0.6471		
CA5A	0.5455			PCNA	0.5641			CA2	1	CPA1	0.5333	PARP1	0.52					GABBR1	0.5882		
CA4	0.5455			SLC7A5	0.5263			CA4	1	CPA3	0.5333	SIRT2	1					GABBR2	0.5882		
CA5B	0.5455			ESR2	0.5625			CA5A	0.5652	CPB1	0.5333							GABRA6	0.5882		
CA6	0.5455			ESR1	0.5625			CA6	1	CPB2	0.5333							GABRR1	0.5882		
CA7	0.5455			CSK	0.6452			CA5B	0.5652	DNPEP	0.5405							GABRQ	0.5882		
CA9	0.6							CA7	1	SLC1A1	0.5							GSTK1	0.5217		
DRD4	0.5116							CA9	1	FOLH1	0.5333							HMGCR	0.5217		
ERAP1	0.5161							FUT7	0.5652	GRIK1	0.5294							SLC22A6	0.7692		
PGR	0.5833							RARB	0.5	KIF11	0.5							SLC22A8	0.7059		
								RARG	0.5	SLC7A5	0.7							SLC6A11	0.5882		
								TOP1	0.5357	LTA4H	0.5128							TLR2	0.7059		

TTR	0.5161	TACR1	0.5588
TYR	0.5217	NOS1	0.5429
		NOS2	0.5429
		NOS3	0.5429
		PPARG	0.6875
		PTPN1	0.6452
		PTPRA	0.5312
		SLC15A1	0.6452
		TAAR1	0.5185
		TPH1	0.5116
		TH	0.5294
